# Productivity and quality-related traits of wheat germplasm affected by heat stress

**DOI:** 10.1371/journal.pone.0333505

**Published:** 2026-04-13

**Authors:** Vahid Rezaei, Mohammad Mahdi Majidi, Aghafakhr Mirlohi, Ghodratollah Saeidi

**Affiliations:** Department of Agronomy and Plant Breeding, College of Agriculture, Isfahan University of Technology, Isfahan, Iran; ICAR Indian Institute of Wheat and Barley Research, INDIA

## Abstract

Heat stress (HS) significantly impedes wheat production, making the development of heat-tolerant cultivars increasingly essential in the context of climate change. This study evaluated 153 elite spring wheat (*Triticum aestivum* L.) genotypes from the Wheat Association Mapping Initiative (WAMI) panel and three controls in field trials conducted during the 2020–2021 and 2021–2022 growing seasons at the Isfahan University of Technology research farm, Iran. Two sowing dates (SD; fall and spring) under full irrigation were employed to replicate HS conditions, with spring SD simulating terminal HS and reflecting regional farming practices. HS reduced days to flowering (DF), anthesis (DA), and maturity (DM) by 36–45%, shortened the grain-filling period (GFP), and decreased grain yield (GY) by ~25%, while key flour-quality traits (e.g., Zeleny index and grain hardness) remained stable under both SDs. Considerable genotypic variability was observed in both agronomic and quality traits. Stress tolerance and sensitivity indices (STI, MP, YSI, and HSI) were used to classify genotypes, with HSI identified as the most effective index due to its strong association with yield performance under HS. Several WAMI lines (e.g., 029, 123, 104, 067, and 139) demonstrated high yield potential combined with robust heat tolerance, as evidenced by their reduced yield loss under HS. These findings highlight the value of the WAMI panel for identifying heat-resilient wheat genotypes and providing critical insights for breeding programs targeting improved wheat performance under terminal HS and water-limited environments.

## 1. Introduction

Heat and drought stress frequently occur alone or in combination in arid and semi-arid regions, severely restricting cereal yields and representing major agricultural constraints [1,2]. Terminal heat stress (HS) is particularly critical for wheat production [3]. Rising temperatures combined with drought intensify food security risks and compromise global wheat supply. During the grain-filling period (GFP), each 1 °C increase above 15 °C can reduce grain yield (GY) by 3–4% under moderate HS (25–32 °C), as demonstrated in both controlled and field studies [4]. Addressing these challenges is essential to meet Sustainable Development Goal (SDG) 2: Zero Hunger [5].

Wheat thrives in mild climates, with optimal growth at 18–22 °C. When temperatures exceed 24 °C during reproductive stages, wheat undergoes HS that markedly reduces grain yield (GY) [6,7]. The most vulnerable periods are flowering and grain filling [8]. In the Isfahan region, Iran, climate records indicate that under spring sowing, maximum daily air temperature often raise above 30 °C in May (flowering; DF) and exceed 35 °C in June (grain filling), whereas fall sowing coincided with milder conditions of about 20–28 °C at the corresponding stages. These thermal contrasts highlight the suitability of staggered sowing dates (SD) for evaluating heat tolerance in the WAMI panel. This vulnerability is concerning, as much of global wheat production already occurs in regions experiencing or projected to face increasing HS [9]. By the end of the 21st century, average global temperature is projected to rise by 1.8–5.8 °C, posing a severe threat to food security [10,11].

However, research has shown that adopting fundamental strategies can mitigate the impact of HS. One key approach is the development of spring wheat varieties through evaluation in diverse environments and identification of heat-tolerant (HT) genotypes [12,13]. Among these strategies, adjusting SD to expose plants to contrasting thermal conditions is particularly effective [14,15], and this approach was also applied in the present study.

The Wheat Association Mapping Initiative (WAMI) panel comprises 287 elite spring bread wheat lines developed by CIMMYT over three decades [16,17]. Prior studies have largely focused on association mapping 16 [16,17], whereas field-based evaluations of heat tolerance across contrasting sowing dates and environments remain limited. Here, we address this gap by evaluating a 153-line subset of WAMI alongside three controls (commercial cultivars) under fall vs spring sowing at the Isfahan University of Technology (IUT) research farm, Isfahan, Iran, to impose terminal HS conditions.

Identifying HT genotypes under field conditions is highly challenging due to the complex, polygenic nature of stress tolerance, which is strongly influenced by genotype (G) × environment (E) interactions [1,18]. GY itself is a complex quantitative trait regulated by multiple components and critically affected by environmental and epigenetic factors. Nonetheless, progress from breeding programs demonstrates the potential of wheat to meet global food demands [19]. These challenges emphasize the need for research targeting G, E, and G × E interactions across diverse environments [20,21]. Trait-based selection methods have been highlighted as essential for sustainable wheat production [22], and the development of stress-tolerant genotypes is also a key strategy for addressing production challenges and malnutrition [22,23]. Among various tools, stress tolerance and sensitivity indices are widely used to evaluate genotypic responses. Yield Stability Index (YSI) [24] and the Stress Tolerance Index (STI) [25] assess genotype stability across environments, while the Heat Susceptibility Index (HSI) is particularly recommended for screening HT wheat lines [26]. Indeed, recent studies have successfully employed HSI to distinguish tolerant genotypes [11,27].

As HS primarily affects reproductive structures and reduces both GY and quality [9], identifying HT wheat varieties has become a breeding priority. In Iran, spring wheat sowing after mid-winter (January–February) is common due to environmental constraints, including seasonal rivers, limited autumn irrigation, low rainfall, and mild winters that allow spring genotypes to grow without vernalization. Thus, the late sowing used in this study not only simulated terminal heat stress but also reflected real agricultural practices under climatic and water-resource limitations. Adjusting SD has been shown to effectively evaluate genotype stability across management practices and aligns with CIMMYT’s breeding objectives [13]. Evaluating the WAMI panel under these conditions provide critical insights for selecting HT genotypes that can directly benefit farmers and serve as genetic resources for future breeding programs. This study is a phenotypic field evaluation; no genomic data are presented. Accordingly, the main objectives of this study were: (1) to evaluate heat tolerance in the WAMI panel under contrasting sowing dates (fall and spring) in the Isfahan region, Iran, benchmarking the genotypes against well-adapted commercial cultivars; (2) to determine the impact of HS on agronomic and grain quality traits in order to identify stable genotypes that combine yield performance with acceptable flour quality; and (3) to identify heat-tolerant (HT) genotypes through phenotypic selection using multiple stress tolerance and sensitivity indices, thereby providing genetic resources for cultivation under HS conditions and for future breeding and genetic improvement.

## 2. Material and methods

### 2.1. Plant genetic materials

The genotypes used in this study were sourced from the International Maize and Wheat Improvement Center (CIMMYT) international nurseries, annually distributed through its Global Genetic Resources Program. The plant material comprised 153 spring bread wheat (*Triticum aestivum* L.) genotypes, carefully selected from the 287-lines wheat association mapping initiative (WAMI) panel, complemented by three control cultivars (Pishtaz, Ghods, and Roshan). The WAMI subset was selected based on pedigree and phylogenetic relationships to ensure broad genetic diversity while minimizing confounding variation in phenology and plant height [16,17,28]. Pedigree information for the Iranian control cultivars and additional details on the WAMI panel are provided in S1 Table in S1 File.

### 2.2. Experimental conditions and sowing management

**Fig 1:**
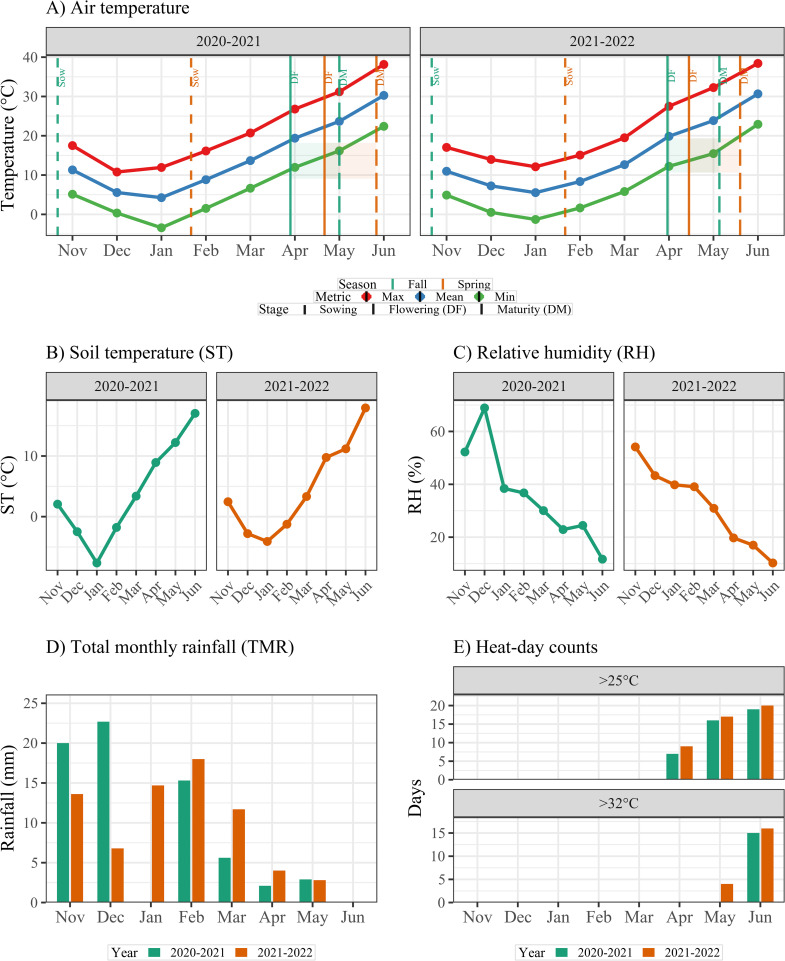
Climatic profile of the experimental site across two growing seasons (2020–2021 and 2021–2022) and two sowing dates (Fall = normal; Spring = heat stress). (A) Maximum, mean, and minimum air temperatures (°C) with phenological markers: sowing (Sow; fixed at 5 November for Fall and 5 February for Spring), flowering (DF), and physiological maturity (DM). The shaded interval denotes the grain‐filling period (DF–DM). (B) Mean soil temperature (ST, °C). (C) Mean relative humidity (RH, %). (D) Total monthly rainfall (TMR, mm). (E) Number of days with air temperature >25 °C (Abo 25; chronic heat) and >32 °C (Abo 32; extreme heat). Data are summarized at monthly resolution from November through June in both seasons.

The experiment was conducted at the Isfahan University of Technology (IUT) research farm, Isfahan, Iran (32°32’N, 51°23’E; 1630 m a.s.l) during the 2020–2021 and 2021–2022 growing seasons. The experimental site has a sandy loam soil, and no fertilizer was applied during the experiment. Seasonal climatic variables (daily air and soil temperature, rainfall, relative humidity) and phenological markers are shown in Fig 1 to illustrate the thermal contrast between SDs and the imposition terminal HS. Genotypes were sown on 5 November (fall; normal conditions) and 5 February (spring; terminal HS) under full irrigation. Fall sowing on 5 November reflects regional practice; earlier October planting is rarely feasible due to limited fall irrigation and field-operation constraints.

The trial followed an alpha-lattice design (12 × 13) comprising 156 genotypes (153 WAMI lines + 3 controls), with two replications within each SD in each year. Within each SD, genotypes were randomized within incomplete blocks for each replication; each replicate comprised 12 incomplete blocks of 13 plots. This design was employed to reduce spatial variability and improve the precision of genotype comparisons. Each plot consisted of two rows, 2.0 m in length, 20 cm between rows, and 60 cm between plots. To minimize border effects, the central 1.0 m of each of the two rows per plot was harvested, yielding a harvested area of 0.40 m^2^ (2 rows × 1.0 m × 0.20 m). Pressurized drip irrigation (lateral spacing 20 cm) was used, and irrigation scheduling was managed to prevent water deficit throughout both seasons, thereby isolating heat effects.

### 2.3. Phenological, morphological, and yield measurements

The traits for days to booting (DB), flowering (DF), anthesis (DA), and maturity (DM) were recorded as the number of days from sowing until 50% of plants in each plot reached; for DB, the criterion was 50% of culms showing booting (flag-leaf sheath swollen), for DF/DA it was 50% of spikes showing visible anthers/onset of anthesis, and DM was recorded at physiological maturity. Plant height (PH), spike length (SL), and peduncle length (PL) were measured in in centimeters (cm) on five randomly selected plants per plot. Thousand-kernel weight (TKW, g) was measured on cleaned grain per plot as the mean of two 500-kernel subsamples, scaled to 1000 kernels, and reported at 14% moisture. Grain yield (GY; g/m^2^) and biological yield (BY; g/m^2^) were computed from the harvested area (0.40 m^2^) and standardized to m^2^ by dividing by 0.40 (i.e., × 2.5). Test weight (TW; kg hL ⁻ ¹) was measured per plot following AACC Method 55−10 [29]. The harvest index (HI) was calculated as [30]:


HI=Gy/By×100

Where, Gy and By denote grain yield and biological yield (g m ⁻^2^), respectively.

### 2.4. Near-Infrared (NIR) analysis of whole wheat flour

Whole-wheat flour was analyzed using an NIR analyzer (Inframatic 8620, Perten Instruments AB, Sweden). The following traits were obtained from manufacturer-supplied calibrations traceable to AACC/ICC reference methods: percentage of grain protein (PGP, %), Zeleny sedimentation volume (ZSV, cm^3^), and grain hardness (GH, %). Values for PGP and ZSV were reported on a 14% moisture basis. Further details of calibration and standardization follow AACC International [31].

### 2.5. Statistical data analysis

The data matrix was prepared using Microsoft Excel 2021, and statistical analyses were conducted in SAS (version 9.4; SAS Institute Inc., Cary, NC, USA) using the general linear model (PROC GLM). Biplots (PCA biplots) were generated using Minitab (version 17) and XLSTAT (version 2016); additional figures were produced in R (version 4.5; R Core Team) using ggplot2. Quality control included field-book cross-checks; outliers were screened via studentized residuals (|rstudent| > 3, preliminary RCBD) and removed only if non-experimental; missing values were handled by complete-case analysis; PCA used standardized z-scores on complete cases.

For across environment analyses, a combined analysis of variance (ANOVA) was performed under a randomized complete block design (RCBD) framework because preliminary checks indicated negligible inter-block effects in the alpha-lattice layout. The model included genotype (G) and sowing date (SD) as fixed effects, with year (Y) and replication nested within Y × SD as random effects. Multiple comparisons of means were conducted using Fisher’s least significant difference (LSD) test (*p* ≤ 0.05). Pearson’s correlation coefficient was used to assess trait relationships.

To evaluate traits and assist in identifying tolerant genotypes, principal component analysis (PCA) was conducted under both normal and terminal HS conditions, and biplots were used to visualize genotype–trait associations.

Broad-sense heritability (h^2^_b_) under normal and HS conditions was calculated as [32]:


$$h^{\text{2}}b=\sigma^{\text{2}}G/\sigma^{\text{2}}P$$

Where, $\sigma^{\text{2}}G$ and $\sigma^{\text{2}}P$ represent the genotypic and phenotypic variances, respectively.

To evaluate heat tolerance and classify genotypes, the following indices were computed:

Heat susceptibility index $(\text{HSI})\;=\;(\text{1}-Y_{S}/\;Y_{P})/(\text{1}-X_{S}/X_{P}$ [33]

Yield stability index $(\text{YSI})\;=Y_{S}/\;Y_{P}$ [24]

Stress tolerance index $(\text{STI})\;=(Y_{S}\times Y_{P})/{\left(X_{P}\right)}^{\text{2}}$ [25]

Tolerance index $(\text{TOL})=Y_{P}-\;Y_{S}$ [34]

Mean productivity $(\text{MP})=(Y_{S}+Y_{P})/\text{2}$ [34]

Geometric mean productivity $(\text{GMP})=\sqrt{\left(Y_{S})\times {(Y}_{P}\right)}$ [25]

Yield Index $(\text{YI})=\;Y_{S}/\;X_{S}$ [35]

Harmonic Mean $\left(\text{HM})=[\text{2}\left(Y_{P}\times Y_{S}\right)]/(\;Y_{P}+Y_{S}\right)$ [36]

Mean relative performance $\left(\text{MRP})\;=(Y_{S}/\;X_{S})+(Y_{P}/\;X_{P}\right)$ [37]

Percent yield Reduction $(\text{PYR})\;=(Y_{P}-\;Y_{S})/\;Y_{P}\times \;\text{1}\text{0}\text{0}$ [38]

$Y_{S}$ denotes grain yield under stress conditions (spring-sown), while $Y_{P}$ refers to grain yield under normal sowing conditions (fall-sown). $X_{S}$ and $X_{P}$ represent the average yields of all genotypes under stress and normal conditions, respectively.

To identify superior HT genotypes, a composite ranking approach was applied using three established indices (HSI, MP, STI). For each genotype, index values were converted to comparable ranks and aggregated by mean rank, prioritizing low HSI in combination with high MP and STI. The top 10 genotypes were thus identified, and the complete ranking of all genotypes is provided in S7 Table in S1 File.

## 3. Results

Combined analysis of variance (Table 1) showed that the effect of SD on DF, DA, DM, and HI traits was significant at P < 0.05, and on the traits of GY and BY at the 0.01 level of probability. Results of separate ANOVA for each sowing date are provided in S4 and S5 Tables in S1 File. In contrast, the traits such as PH, PL, and SL were not affected by HS. These results seem reasonable given the type of population used in this study, as the WAMI panel is a modified population based on morphological traits and plant height [16,39]. The effect of year (Y) on the traits was not significant; however, the interaction effect of Y × SD was significant for all traits except for DB, PGP, ZSV, and GH. Significant differences were also observed among the genotypes for all measured traits at P < 0.01. Additionally, the interaction effect of G × SD was significant at P < 0.01 for all the traits except for DF, SL, PGP, and ZSV.

**Table 1:** Results of the combined analysis of variance (RCBD) for phenological, morphological, yield, and quality traits in the WAMI panel (153 genotypes) and three control cultivars, across two sowing dates (SD: Fall = normal; Spring = heat stress) over two years.

Traits	df	Means of squares							
		DB	DF	DA	DM	PH	PL	SL	TKW
Year(Y)	1	6264^ns^	735^ns^	812^ns^	303^ns^	957^ns^	70.5^ns^	25.9^ns^	194^ns^
Sowing Date (SD)	1	163030^ns^	1635458^*^	1060791^*^	159377^*^	49029^ns^	1183^ns^	151.8^ns^	11684^**^
SD*Y	1	16197^**^	5243^**^	1274^**^	8375^**^	1954^**^	272.8^*^	31^**^	6.92^ns^
Rep (SD*Y)	4	19.66	11	8.52	4.06	24.0	31.3	1.28	10.6
Genotype (G)	155	20.03^**^	19.04^**^	12.40^**^	9.13^**^	151.8^**^	34.3^**^	3.56^**^	118^**^
G*SD	155	10.70^**^	5.10^ns^	3.55^**^	9.15^**^	32.1^**^	10.2^**^	0.68^ns^	5.43^**^
G*Y	155	9.0^**^	6.49^ns^	1.94^ns^	5.14^ns^	32.8^ns^	7.24^ns^	0.56^ns^	1.81^**^
G*SD*Y	155	5.96^**^	5.82^**^	2.01^**^	6.02^**^	21.4^**^	6.39^**^	0.53^**^	1.16^ns^
Residual	620	1.47	0.91	0.78	1.25	11.9	3.89	0.29	3.27
R^2^		0.99	0.99	0.99	0.99	0.92	0.81	0.85	0.93
CV (%)		1.30	0.77	0.67	0.71	4.31	7.32	6.46	4.60
**Traits**	**df**	**Mean of squares**							
		**TW**	**GY**	**BY**	**HI**	**PGP**	**ZSV**	**GH**	
Year (Y)	1	84.1^ns^	1472763^ns^	17671976^ns^	9.15^ns^	1.63^ns^	3.43^ns^	5.28^ns^	
Sowing Date (SD)	1	1621^ns^	15800400^**^	5069145^**^	16133^*^	8.67^ns^	141.6^ns^	438^ns^	
SD*Y	1	97.1^**^	3513^ns^	113^ns^	74.9^**^	57.4^*^	14.5^ns^	297^*^	
Rep (SD*Y)	4	43.2	22362	80419	7.94	4.25	13.2	25.2	
Genotype (G)	155	20.3^**^	57462^**^	286975^**^	40.4^**^	0.78^**^	1.70^**^	8.22^**^	
G*SD	155	11^**^	18210^**^	262751^**^	20.2^**^	0.22^ns^	0.57^ns^	4.73^**^	
G*Y	155	4.24^ns^	10713^ns^	101416^ns^	4.84^ns^	0.17^ns^	0.48^ns^	1.35^ns^	
G*SD*Y	155	3.50^ns^	12049^**^	129018^**^	5.59^**^	0.21^**^	0.71^**^	1.64^**^	
Residual	620	2.96	2358	16465	1.87	0.091	0.26	0.72	
R^2^		0.81	0.95	0.93	0.95	0.84	0.81	0.88	
CV (%)		2.07	6.17	4.84	4.61	2.32	0.74	1.95	

R^2^ = coefficient of determination; CV (%) = coefficient of variation; * and ** indicate significance at p < 0.05 and p < 0.01, respectively. ns: non-significant. Trait abbreviations: Days to booting (DB), Days to flowering (DF), Days to anthesis (DA), Days to maturity (DM), Plant height (PH), Peduncle length (PL), Thousand Kernel Weight (TKW), Spike length (SL), Test weight (TW), Grain yield (GY), Biological yield (BY), Harvest index (HI), Percentage of grain protein (PGP), Zeleny sedimentation volume (ZSV), Grain hardness (GH).

### 3.1. The effect of heat stress (HS) on the measured traits

The occurrence of HS due to late SD (spring) significantly impacted most of the measured traits, resulting in their reduction (Table 2). The most substantial decreases were observed for DF, DM, DA, and DB phenological traits, with their reductions of 45.4%, 36.9%, 36.3%, and 21.9%, respectively. This decline in phenological traits shortened the GFP, leading to a 25% decrease in GY compared to normal sowing conditions. Considering that air temperatures exceeded 30 °C during flowering and 35 °C during grain filling, the imposed HS can be classified as moderate to severe. Additionally, yield-related traits such as BY, HI and TKW showed a reduction of 4.71%, 21.6% and 14.4% respectively. In contrast, PH and height-related traits such as PL and SL showed no significant reductions, and this pattern was consistent across years and replications. This can be explained by the fact that these traits are largely determined during earlier developmental stages and because the WAMI panel was pre-selected for reduced variation in PH and morphology [16,39,40]. Moreover, HS also caused declines in PGP, ZSV, and GH; however, these effects were not statistically significant.

**Table 2:** Means, standard deviation, and broad-sense heritability (h2_b_) of phenological, morphological, yield, and quality traits in the WAMI panel (153 genotypes) and three control cultivars grown under two sowing dates (Fall = normal condition; Spring = heat stress condition).

Trait	Normal condition			Heat stress condition			Mean reduction (%)
	Mean	(%) h^2^_b_	Std	Mean	(%) h^2^_b_	Std		LSD (0.05)
DB	104.5^a^	2.24	47	81.6^a^	2.82	28	91.5	21.9
DF	159.5^a^	2.16	49	87.1^b^	2.30	33	52.1	45.4
DA	161^a^	1.89	57	102.5^b^	1.49	81	25.7	36.3
DM	193.5^a^	1.97	61	122^b^	2.03	32	65.8	36.9
PH (cm)	86.4^a^	5.85	13	73.89^a^	5.90	19	31.8	14.5
PL (cm)	27.9^a^	2.73	35	25.97^a^	3.33	84	11.9	6.91
SL (cm)	8.69^a^	0.86	88	7.99^a^	0.93	88	4.00	8.05
TKW (gr)	42.4^a^	4.14	86	36.3^b^	4.20	94	1.82	14.39
TW (kg/hl ⁻ ¹)	84.3^a^	2.17	86	82^a^	2.82	36	7.08	2.72
GY(g/m^2^)	899^a^	129.2	np	674^b^	99.6	np	42.6	25.0
BY(g/m^2^)	2713^a^	330	np	2585^b^	315	np	7.65	4.71
HI (%)	33.3 ^a^	3.74	35	26.1^b^	2.38	56	6.22	21.6
PGP (%)	13.06^a^	0.44	96	12.90^a^	0.51	95	5.45	1.22
ZSV (mL)	32.47^a^	0.78	84	31.80^a^	0.76	90	2.74	2.06
GH (%)	44.01^a^	0.89	81	42.83^a^	1.99	66	12.4	2.68

LSD (0.05) = least significant difference at the 5% probability level. h^2^b = broad-sense heritability. Trait abbreviations: Days to booting (DB), Days to flowering (DF), Days to anthesis (DA), Days to maturity (DM), Plant height (PH), Peduncle length (PL), Thousand Kernel Weight (TKW), Spike length (SL), Test weight (TW), Grain yield (GY), Biological yield (BY), Harvest index (HI), Percentage of grain protein (PGP), Zeleny sedimentation volume (ZSV), Grain hardness (GH). Non-predictive (np).

### 3.2. Exploring principal component analysis (PCA) and trait associations

**Fig 2:**
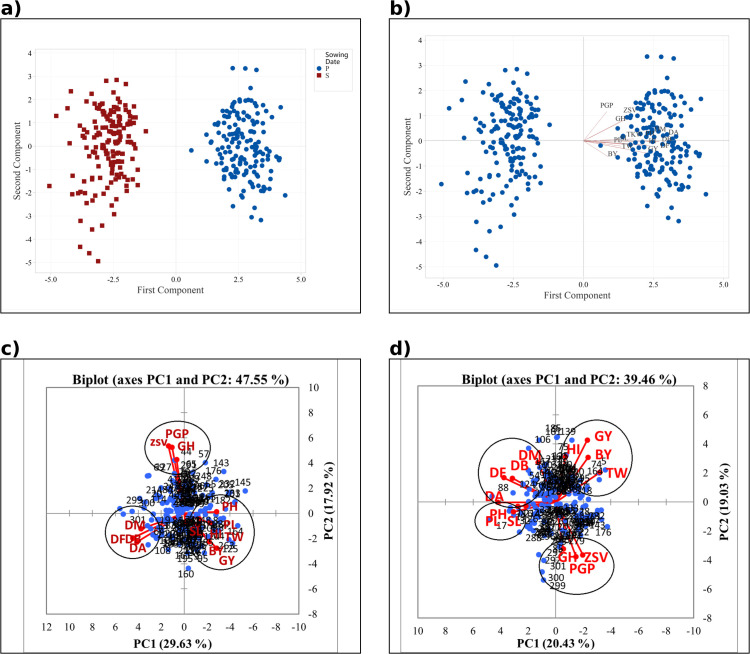
Principal component analysis (PCA) of phenological, morphological, yield, and quality traits in the WAMI panel (153 genotypes) and three control cultivars under two sowing dates (Fall = NC; Spring = HS). (a, b) show the score plot of genotypes and the corresponding trait biplot across both SD, respectively. (c, d) present trait biplots under Fall (NC) and Spring (HS) sowing, respectively. traits: days to booting (DB), days to flowering (DF), days to anthesis (DA), days to maturity (DM), plant height (PH), peduncle length (PL), spike length (SL), test weight (TW), biological yield (BY), grain yield (GY), harvest index (HI), Thousand Kernel Weight (TKW), Zeleny sedimentation volume (ZSV), Grain hardness (GH), and percentage of grain protein (PGP).

The PCA analysis conducted under both normal (NC) and HS conditions revealed a clear separation of genotypes in these two environments (Fig 2a and 2b). Most measured traits loaded positively on PC1, with phenological traits (DF, DA, DM, DB) contributing strongly under NC, while yield-related traits (GY, BY, HI, TKW) showed higher loadings under HS. To gain more detailed insights, PCA was also conducted separately for each SD (Fig 2c and 2d; Table 3).

**Table 3:** Correlation coefficients among phenological, morphological, yield, and quality traits in the WAMI panel (153 genotypes) and three control cultivars under heat stress conditions (above the diagonal, Spring SD) and normal conditions (below the diagonal, Fall SD).

Traits	DB	DF	DA	DM	PH	PL	SL	TW	GY	BY	HI	PGP	ZSV	GH	TKW
**DB**		0.6**	0.43**	0.35**	-0.05	0.06	0.05	-0.03	-0.07	-0.11	0.03	-0.11	-0.14	-0.13	0.12
**DF**	0.98**		0.57**	0.39**	-0.01	0.13	0.14	-0.27**	-0.01	-0.01	0.01	-0.07	-0.2**	-0.02	0.04
**DA**	0.83**	0.81**		0.02	-0.01	0.11	0.18*	-0.37**	-0.27**	-0.34**	0	-0.16*	-0.2**	-0.06	-0.05
**DM**	0.7**	0.69**	0.77**		0.05	0.04	0.02	0.17*	0.18**	0.16*	0.1	-0.09	-0.02*	-0.06	0.11
**PH**	-0.4**	-0.4**	-0.3**	-0.15*		0.8**	0.5**	-0.14	0.004	0.03	-0.04	-0.08	-0.04	-0.01	-0.11
**PL**	-0.16*	-0.18*	-0.03	0.11	0.57**		0.54**	-0.25**	-0.11	-0.05	-0.11	-0.06	-0.04	-0.01	-0.13
**SL**	0.03	0.02	0.11	0.22**	0.33**	0.44**		-0.34**	0.03	-0.01	0.06	-0.01	-0.01	0.03	-0.1
**TW**	-0.3**	-0.2**	-0.3**	-0.2**	0.27**	0.06	-0.03		0.31**	0.3**	0.11	-0.07	0.06	-0.11	0.03
**GY**	-0.3**	-0.3**	-0.15	-0.25**	0.29**	0.19*	0.26**	0.26**		0.81**	0.6**	-0.13	-0.13	-0.02**	0.05
**BY**	-0.22**	-0.24**	-0.09	-0.1	0.39**	0.18*	0.13	0.24**	0.63**		0.04	-0.04	-0.06	-0.04	-0.06
**HI**	-0.12	-0.13	-0.08	-0.2**	-0.03	0.04	-0.01	0.07	0.57**	-0.28**		-0.15*	-0.11	-0.25**	0.02
**PGP**	-0.01	-0.01	-0.01	0.02	-0.1	-0.09	-0.05	-0.13	-0.22**	-0.23**	-0.02		0.76**	0.54**	0.08
**ZSV**	0.03	-0.02	0.03	0.15	-0.01	0.02	0.03	-0.13	-0.35**	-0.25**	-0.15	0.8**		0.29	-0.05
**GH**	-0.01	-0.03	-0.01	0.09	0.11	0.08	0.1	-0.1	-0.15*	-0.06	-0.12	0.52**	0.58**		0.06
**TKW**	0.12	0.11	0.11	0.05	-0.07	-0.01	0.03	0.08	0.18	0.01	0.02	0.06	0.02	0.1	

* and ** indicate significant at p < 0.05 and p < 0.01, respectively.

DB: Days to booting, DF: Days to flowering, DA: Days to anthesis, DM: Days to maturity, PH: Plant height, PL: Peduncle length, SL: Spike length, TW: Test weight, GY: Grain yield, BY: Biological yield, HI: Harvest index, PGP: Percentage of grain protein, ZSV: Zeleny sedimentation volume, GH: Grain hardness, Thousand Kernel Weight (TKW).

Under NC phenological traits (DF, DA, DM, DB) were tightly correlated, whereas GY clustered with BY and HI, and quality traits (PGP, ZSV, GH) formed a distinct group. Under HS, GY maintained strong associations with BY and HI (r = 0.81 and r = 0.60, respectively), while correlations with PH, PL, and SL were weak (r < 0.3) and not considered further. Quality traits also remained strongly correlated (PGP with ZSV and GH), indicating a stable relationship across environments. Notably, under NC, GY showed significant negative correlations with both phenological and quality traits, whereas these relationships diminished under HS, suggesting altered allocation between yield and quality under stress. The biplots further highlighted specific genotypes: under NC, lines 3, 26, 125, and 164 exhibited high yield potential, while lines 44, 57, 69, 127, and 292 showed higher protein content. Under HS, genotypes 5, 74, and 164 maintained high yields, whereas 292, 295, and the three controls (299, 300, 301) were distinguished by higher protein content. These patterns demonstrate how PCA effectively separated genotype performance under contrasting environments.

### 3.3. Measurement of stress tolerance and sensitivity indices

**Fig 3:**
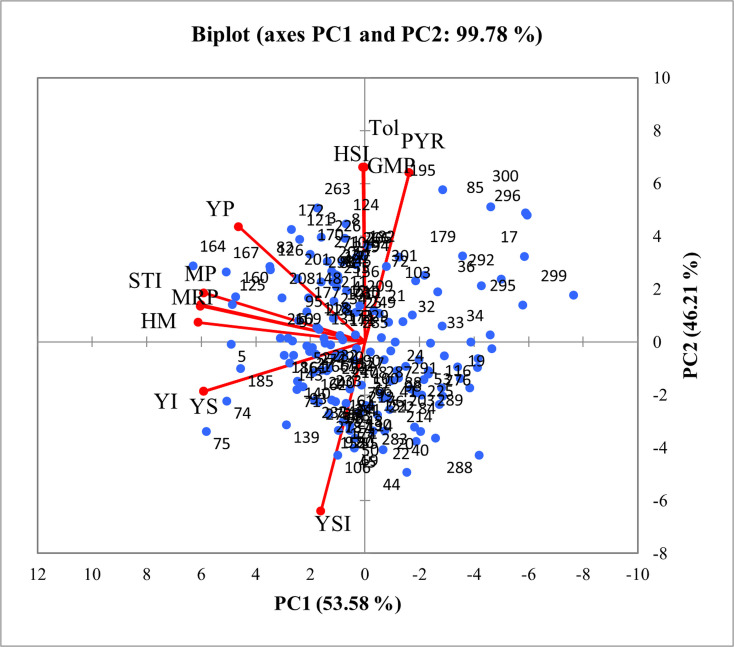
Principal component analysis (PCA) biplot (PC1 vs PC2) of stress tolerance and sensitivity indices in the WAMI panel (153 genotypes) and three control cultivars under normal (Fall = NC) and heat stress (Spring = HS) conditions. Indices: Yp: Grain yield of genotypes under normal condition, Ys: Grain yield of genotypes under heat stress condition, TOL: Tolerance index, YSI: Yield stability index, HSI: Heat susceptibility index, MP: Mean productivity, GMP: Geometric mean productivity, STI: Stress tolerance index, HM: Harmonic mean, YI: Yield index, MRP: Mean relative performance, PYR: Percent yield reduction.

Following the PCA of phenotypic traits, stress tolerance and sensitivity indices based on GY were calculated under both NC and HS conditions (S2 Table in S1 File). To better explore their interrelationship and to classify genotypes, a PCA biplot of indices (Fig 3) and their correlation matrix (Table 4) were analyzed. The results showed that Y_P_ was positively and significantly correlated with almost all indices. In contrast, Y_S_ was negatively and correlated with Tol, GMP, HSI, and PYR, but positively correlated with STI, YI, HM, YSI, MP, and MRP. Notably, HSI had a very strong positively correlated with Tol, GMP, and Y_P_, and a strong negative correlation with Y_S_, YSI, PYR, and YI. These relationships indicate that HSI can be effectively used to classify genotypes into tolerant, semi-tolerant, and susceptible groups.

**Table 4:** Correlation coefficients among the Stress Tolerance and Sensitivity Indices (Yp, Ys, TOL, YI, YSI, HSI, MP, GMP, STI, MRM, HM, and PYR) under normal (fall SD) and heat stress (spring SD) conditions in the WAMI panel (153 genotypes) and three control cultivars.

Variables	YP	YS	Tol	GMP	MP	YSI	HSI	STI	YI	HM	MRP	PYR
**YP**												
**YS**	0.53^**^											
**Tol**	0.66^**^	−0.28^**^										
**GMP**	0.66^**^	−0.27^**^	0.99^**^									
**MP**	0.91^**^	0.84^**^	0.29	0.29								
**YSI**	−0.43^**^	0.52^**^	−0.96^**^	−0.96^**^	−0.02							
**HSI**	0.43^**^	−0.52^**^	0.96^**^	0.96^**^	0.02	−1.0^**^						
**STI**	0.87^**^	0.88^**^	0.22^**^	0.22^**^	0.99^**^	0.05	−0.05					
**YI**	0.53^**^	1.0^**^	−0.28^**^	−0.27^**^	0.84^**^	0.52^**^	−0.52^**^	0.88^**^				
**HM**	0.82^**^	0.92^**^	0.12	0.13	0.98^**^	0.15	−0.15	0.99^**^	0.92^**^			
**MRP**	0.87^**^	0.88^**^	0.21^**^	0.22^**^	1.0^**^	0.06	−0.06	1.0^**^	0.88^**^	0.99^**^		
**PYR**	0.43^**^	−0.52^**^	0.96^**^	0.96^**^	0.02	−1.0^**^	1.0^**^	−0.05	−0.52^**^	−0.15	−0.06	

* and ** indicate significance at p < 0.05 and p < 0.01, respectively.

Yp: Grain yield of genotypes under normal condition, Ys: Grain yield of genotypes under heat stress condition, TOL: Tolerance index, GMP: Geometric mean productivity, MP: Mean productivity, YSI: Yield stability index, HSI: Heat susceptibility index, STI: Stress tolerance index, YI: Yield Index, HM: Harmonic Mean, MRP: Mean relative performance, PYR: Percent yield Reduction.

### 3.4. Identification of heat-tolerant (HT) genotypes using the heat susceptibility index (HSI)

**Fig 4:**
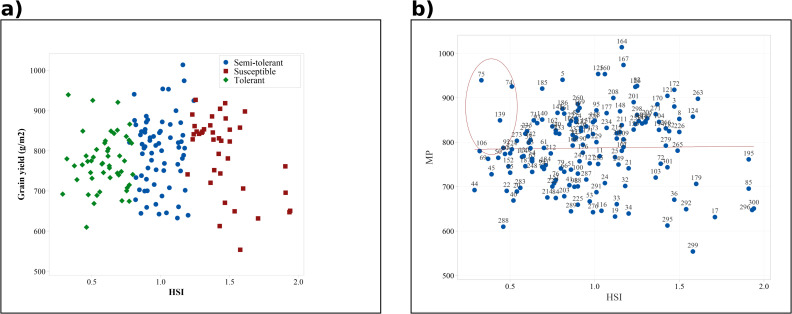
Classification and selection of genotypes based on heat susceptibility index (HSI). (a) Genotypes grouped as Tolerant (HSI ≤ 0.8), Semi-tolerant (0.8 < HSI ≤ 1.3), and Susceptible (HSI > 1.3). (b) Scatter plot of HSI versus MP (Mean Productivity), highlighting genotypes that combine low susceptibility with higher yield across normal (Fall SD) and heat stress (Spring SD) conditions in the WAMI panel (153 genotypes) and three control cultivars.

To identify HT genotypes within the studied population, HSI values were calculated for all genotypes based on previous findings and corroborating studies from other researchers. These values classified the genotypes into three categories: Tolerant (HSI ≤ 0.8), Semi-tolerant (0.8 < HSI ≤ 1.3), and Susceptible (HSI > 1.3) (S3 Table in S1 File). In total, 10 genotypes were categorized as Tolerant, 18 as Semi-tolerant, and 9 as Susceptible. On average, tolerant genotypes maintained higher GY across environments, while susceptible ones showed substantial reductions under HS. Representative tolerant genotypes included 22, 44, 45, 50, and 69, whereas genotypes 292 and 296 exemplified the susceptible group. The distribution of genotypes across HSI classes is illustrated in Fig 4a, and the complete list is provided in S3 Table in S1 File. Notably, the three control cultivars used in this study were also categorized as Susceptible. To further refine selection, a regression analysis of HSI against MP was performed (Fig 4b), which identified genotypes 69, 74, 75, 106, and 139, as combining higher MP with lower HSI values.

### 3.5. Identification of heat-tolerant (HT) genotypes based on selection indices

Ten genotypes were identified as the top HT candidates, with superior performance across the three indices (HSI, MP, STI). These genotypes combined high productivity under both NC and HS conditions with low susceptibility and favorable tolerance indices. Table 5 lists the top 10 genotypes, while S6 Table in S1 File. provides mean values of GY, TKW, and PGP for these genotypes under both conditions with LSD-based mean separations.

**Table 5:** Top 10 heat-tolerant genotypes from the WAMI panel, selected based on the average ranks of HSI, MP, and STI indices.

Rank	Genotype	WAMI ID	YP	YS	HSI	MP	STI
1	75	104	979	898	0.33	939	1.09
2	74	123	988	862	0.51	925	1.05
3	139	105	898	800	0.44	849	0.89
4	185	292	1007	833	0.69	920	1.04
5	5	039	1046	834	0.81	940	1.08
6	140	006	931	771	0.69	851	0.89
7	71	271	923	775	0.64	849	0.88
8	186	058	974	776	0.81	875	0.93
9	143	204	959	772	0.78	866	0.92
10	93	035	919	766	0.66	842	0.87

Yp: Grain yield of genotypes under normal conditions (Fall SD); Ys: Grain yield of genotypes under heat stress conditions (Spring SD); HSI = Heat susceptibility index; MP = Mean productivity; STI = Stress tolerance index. Mean Rank represents the average ranking of each genotype across the three indices (HSI, MP, STI). Lower HSI values and higher MP and STI values indicate superior heat tolerance.

## 4. Discussion

HS during the reproductive stages of crop growth can significantly impact reproductive processes in plants, including pollen production and maturation, as well as embryo formation and development [9,41]. Moreover, HS can shorten wheat’s GFP, reduce the crop cycle duration, and ultimately lead to significant declines in GY [8]. In our study, by adjusting the SD, we exposed the plants to HS during critical reproductive growth stages. However, it is important to note that not all wheat cultivars respond uniformly to HS. Findings of numerous studies emphasize that the genetic diversity present in wheat populations is a valuable resource for identifying HT genotypes, which is crucial for sustainable production [42–44]. The results of this study showed that the spring SD significantly reduced GY and other measured traits, likely due to exposure of plants to HS. This finding is consistent with previous studies that highlight wheat’s sensitivity to terminal HS [45]. Moreover, the ANOVA results indicated significant effects of genotype (G) and the G × SD interaction, reflecting considerable genetic diversity among the genotypes and their varying responses to changes in SD. This provides an opportunity to identify and select HT genotypes, as genetic variation is essential for selection [18,43,46]. The significant G × SD interaction observed in this study highlights the importance of evaluating genotypes across contrasting SDs. Such interactions provide opportunities to identify broadly adapted genotypes that combine stability under NC with resilience under HS, which is a crucial strategy for breeding programs targeting diverse and unpredictable environments*.*

Trait comparisons under NC and HS conditions revealed that HS significantly reduced all measured phenological traits. However, PH, PL, and SL were comparatively less affected. This suggests that applying HS during the reproductive stages of growth primarily spared the vegetative phases from stress exposure. The genotypes used from the WAMI panel were specifically semi-dwarf and improved genotypes [40,47,48]. Despite this, HS significantly shortened the flowering, anthesis, and maturity periods. GY is influenced by its components and determined through various pathways, with the GFP being particularly critical [49]. Thus, the reduction in these phenological traits due to HS caused a substantial impact on GY. Our study found that GY and its related traits were significantly reduced under HS, which is in agreement with previous findings [1,50]. Quantitative traits like GY are complex and influenced by G × E interactions, necessitating further evaluation across diverse environments [12,18,19]. This prompted us to select genotypes with higher GY and stability under HS from a relatively large population. Langridge and Reynolds [8] reported that selecting varieties that perform well under poor climate conditions is the most reliable approach for achieving broad adaptation and optimal performance under adverse conditions. Thus, developing heat-resistant spring wheat varieties with stable GY and quality is crucial and economical for food security [13]. Although our study did not directly investigate the physiological mechanisms of HT, tolerant genotypes likely possess adaptive mechanisms. These may include increased antioxidant enzyme activity, enhanced cooling systems in the plant, or altered membrane fatty acid composition, all of which could improve plant performance under HS [51]. Future research should further explore these mechanisms in diverse environmental conditions [3] and also focus on quality traits like flour quality and baking characteristics, which are important for processing in food industries.

It is crucial to consider both yield and crop quality simultaneously in breeding programs, particularly under HS conditions [13,14]. A key quality trait of wheat is PGP, which plays a major role in determining bread-making [51,52]. In this study, the spring sowing treatment was not only a strategy to simulate HS but also mirrored actual farming practices in many regions of world such as Iran, where late sowing is common due to depleted water resources, limited autumn rainfall, seasonal rivers, and mild winters that reduce the vernalization requirement. This approach was therefore both agronomically relevant and realistic. Each genotype was sown in two one-meter-long rows per replicate a practical layout that balanced phenotyping efficiency with the limitations of space, time, and resources. Our results showed that SD had no significant effect on PGP, ZSV, and GH, whereas the effects of G and G × SD × Y interaction were significant, indicating genotype-dependent variation in quality traits under varying environmental conditions. The lack of significant differences in PGP, ZSV, and GH under HS may reflect genotype buffering capacity and the protein dilution effect*.* Mahdavi et al. [54] demonstrated that delayed sowing-induced HS in wheat resulted in lighter, smaller grains with a reduced starchy endosperm (characterized by lower amylose content, indicating diminished quality) and elevated PGP under HS conditions compared to normal growing environments [54]. Although total protein content remains a major determinant of wheat quality [55], the type of proteins and other properties, such as GH, also play key roles in defining end-use quality [53–55]. Langridge and Reynolds [8] similarly noted that while severe stress may reduce starch accumulation, even moderate HS can shift the balance between glutenin and gliadin, thereby affecting baking quality [8]. In our study, despite a ~ 25% reduction in GY, the stability of PGP, ZSV, and GH may be partly explained by the significant reduction in TKW. This indicates that smaller grains with a reduced starchy endosperm buffered the relative protein concentration and quality indices. Kernel diameter and kernel width were not measured; however, TKW provides supporting evidence for this interpretation. Although the present study did not explore protein components, our PCA results demonstrated that quality traits under NC differed from those under HS conditions. Under NC, PGP, ZSV, and GH exhibited a significant negative correlation with GY, while under stress conditions, they exhibited a significant negative correlation with phenology traits. These results support the argument that stress conditions shorten the crop cycle, prevent starch deposition, reduce starch content, and may cause underestimation of protein content.

Various strategies have been developed by implementing breeding programs to enhance stress tolerance in different species [3,8,12]. Additionally, a range of statistical methods, including PCA, is utilized to analyze stress effects and compare data across various environments. PCA effectively demonstrates the contribution of each trait, their correlations, genotype distribution in a three-dimensional space, and the role of each principal component (PC) in explaining variations [56]. In our study, PCA for both SD revealed that this strategy successfully differentiated between genotypes, with all measured traits positively correlating with PC1 (Fig 2a and 2b), supporting our research hypotheses. PC1 and PC2 together accounted for a substantial portion of the total variation. Positive correlations between traits, as illustrated by PCA for each SD (Fig 2c and 2d) and correlation analysis (Table 3), indicate that GY can be enhanced through other quantitative traits, thereby improving overall performance. These findings are consistent with those of Fleitas et al. [13], while Guzmán et al. [57] noted that such results enable simultaneous selection for yield and grain quality, leading to higher production with appropriate grain quality [57]. Correlation analysis demonstrated a significant positive association between GY and PH, PL, and SL under NC. However, under HS conditions, there was no significant correlation between these traits and GY, although a significant positive correlation with TKW was observed. This supports our hypothesis and previous findings, showing that HS at the end of the growing season impacts GY more through disturbances in reproductive processes and reductions in TKW than through reductions in PH. Thus, research projects evaluating genotype panels under adverse conditions will be more reliable when the confounding effects of phenological traits and PH are controlled [58].

Given that GY is a highly complex trait influenced by environmental factors, selection strategies often rely on field evaluation with varying conditions and assessing yield stability across different environments [20]. Alternatively, selection can be done based on those related traits with high heritability [21]. However, these strategies are both time-consuming and costly. To reduce time and costs, breeding programs aim to identify a limited number of locations or experimental conditions, such as SD, that effectively represent diverse performance [21]. In many countries, national variety trials can help identify the most suitable environments for evaluating germplasm and make it available to breeders [8,22]. Therefore, this study aimed to select HT genotypes from the WAMI panel, which exhibit more stable performance under both NC and HS conditions in the Isfahan province, Iran. Additionally, to compare the HT level of this population with commonly used commercial cultivars, three Iranian cultivars (Roshan, Ghods, and Pishtaz) were used as controls. The present study found acceptable levels of *h*^*2*^_*b*_ for the measured traits (Table 2). Our findings also emphasize that the genetic diversity within the examined germplasm can be a valuable resource for identifying HT genotypes. Introducing HT genotypes is a crucial strategy for coping with climate change and enhancing food security [12,59].

To develop an effective selection program in plant breeding, it is crucial to employ indices that reliably assess yield stability and identify genotypes with consistent performance under Stress condition. Several indices have been developed specifically for this purpose, with some being more frequently utilized by researchers for evaluating HS [18,59,60]. This study used the indices YP, YS, TOL, GMP, MP, YSI, HSI, STI, YI, MRP, and PYR to identify and select HT genotypes. We used mean GY to calculate these indices to assess heat tolerance in the studied genotypes. These indices facilitated the identification of high-yielding, low-yielding, tolerant, and susceptible genotypes. However, our goal was to identify the most effective single index for genotype screening. A detailed examination of the PCA results and the relationships between the indices revealed a strong negative correlation between YSI and HSI. Furthermore, HSI exhibited a significant positive correlation with Y_P_, GMP, PYR, and TOL, and a significant negative correlation with Y_S_. Based on these findings, corroborated by similar studies, and considering the distribution of measured indices in the biplot space derived from PCA analysis, the HSI index was ultimately chosen for screening and identifying tolerant (HSI ≤ 0.8), semi-tolerant (0.8 < HSI ≤ 1.3), and susceptible (HSI > 1.3) groups. Although STI and MP also showed positive correlations with GY, they were less effective in discriminating tolerant from susceptible genotypes. In contrast, HSI provided a clearer separation and was therefore prioritized as the most reliable index. Lamba et al. [59] reported that genotypes with lower HSI and TOL values are more tolerant and preferable, as they maintain better performance under NC and exhibit less yield reduction under stress. Similarly, Kumar et al. [18] indicated that HSI and YSI are among the most effective indices for identifying stress-tolerant genotypes. Consequently, in this study, the HSI index effectively distinguished tolerant genotypes from susceptible ones. This suggests that HSI serves as an efficient tool for screening genotypes under stress condition. Based on their HSI values, the genotypes were grouped into three categories: tolerant, semi-tolerant, and susceptible (Fig 4 and S3 Table in S1 File). The Iranian genotypes used as controls were also categorized as susceptible. To further identify genotypes that were both HT (with low HSI values) and had high mean productivity under both HS and NC (with high MP values), a regression analysis of MP against HSI was conducted. The tolerant genotypes identified in this study can serve as a genetic resource for breeding programs to develop new wheat varieties with high yield and heat tolerance. Moreover, these genotypes can be utilized as genetic materials in physiological and molecular studies to explore the mechanisms of heat tolerance.

The integration of multivariate analyses, trait correlations, and stress indices enabled a thorough assessment of the WAMI panel under terminal HS. Notably, several genotypes outperformed the Iranian commercial cultivars, which were categorized as susceptible. These results underscore the potential of the WAMI panel as a valuable source of HT germplasm tailored to Iran’s agricultural and climatic conditions. The selected genotypes represent promising resources for breeding programs targeting resilience to climate change. Moreover, the insights gained from this study lay a strong foundation for future advancements in marker-assisted selection, physiological research, and the development of elite wheat varieties with stable, high yields under stress conditions.

## 5. Conclusion

Terminal heat stress (HS) during the reproductive stages of wheat growth significantly reduces productivity and threatens food security. In this study, delayed sowing, simulating terminal HS, caused an average 25% reduction in GY and strongly affected phenological traits and yield components. In contrast, traits related to plant architecture (PH, PL) and flour quality (ZSV, GH) were comparatively less impacted, indicating their relative stability under stress. The observed genetic variability among genotypes and significant G × SD interactions confirmed the potential of the WAMI panel for identifying HT lines. The identification of HT genotypes was accomplished using multiple tolerance and sensitivity indices, with HSI proving to be a reliable tool for classification. Several genotypes from the WAMI panel outperformed local Iranian controls under both NC and HS conditions, making them valuable candidates for further breeding and physiological investigations. This research provides a strong foundation for future studies aiming to develop resilient wheat cultivars adapted to climate change, particularly in regions such as Iran where late sowing is becoming increasingly common due to water scarcity and shifting rainfall patterns. Further work should investigate the physiological and genetic mechanisms underlying heat tolerance, and incorporate detailed grain quality parameters (e.g., protein).

## Supporting information

S1 File **S1 Table.** Additional information on the *Wheat Association Mapping Initiative* (WAMI) population. This population was introduced by the International Maize and Wheat Improvement Center (CIMMYT) in 2009 and comprises 287 advanced elite lines. Based on the recommendations of CIMMYT researchers and experts, a sub-population of 170 lines was selected to ensure the maintenance of the desired level of genetic diversity. This sub-population is labeled as group **S170**. Among these 170 lines, those highlighted in green were utilized for the heat stress study, along with three Iranian cultivars listed at the end of the table. The genotypes were used with their respective names, which are provided in the first column. The pedigree information and selection history of the genotypes are presented in the columns labeled “Cross” and “Selection History,” respectively. **S2 Table.** Values of stress tolerance and sensitivity indices for all studied genotypes. The genotypes included in this study comprise a sub-population of the Wheat Association Mapping Initiative (WAMI), consisting of 153 advanced elite lines along with three Iranian cultivars used as controls (Pishtaz = 299, Qhods = 300, and Roshan = 301). The Gen column lists the genotypes, with their pedigree information provided in S1 Table of this supplementary material. **S3 Table.** Clustering of genotypes from the WAMI sub-population and control cultivars (additional genotype information is provided in S1 Table) into three groups: tolerant, semi-tolerant, and susceptible. Genotypes with HSI values < 0.8 were classified as tolerant, those with values between 0.8 and 1.2 as semi-tolerant, and those with values > 1.2 as susceptible genotypes. In several studies, other researchers have also utilized the HSI index to identify tolerant genotypes, and the results of our study align with their findings. **S4 Table.** Analysis of variance (ANOVA) for agronomic and quality-related traits in the Wheat Association Mapping Initiative (WAMI) panel (153 genotypes and three control cultivars) evaluated under the fall sowing date (SD, normal environment). **S5 Table.** Analysis of variance (ANOVA) for agronomic and quality-related traits in the Wheat Association Mapping Initiative (WAMI) panel (153 genotypes and three control cultivars) evaluated under the spring sowing date (SD, heat-stress environment). **S6 Table.** Mean values of grain yield (GY), thousand-kernel weight (TKW), and protein content (PGP) for the top 10 heat-tolerant genotypes in the Wheat Association Mapping Initiative (WAMI) panel (153 genotypes and three control cultivars) evaluated under normal (Fall SD) and heat-stress (Spring SD) environments, with LSD (0.05) mean separations. **S7 Table.** Complete ranking of 156 wheat genotypes (153 WAMI lines plus three control cultivars) from the Wheat Association Mapping Initiative (WAMI) panel, evaluated under normal (NC; fall sowing) and heat-stress (HS; spring sowing) conditions, using a composite ranking approach based on three indices (HSI, MP, STI).
(DOCX)

S2 File Supplimentary-data. (XLSX)
