# Supplementary material for: Productivity and quality-related traits of wheat germplasm affected by heat stress
Source: PLoS One. 2026 Apr 13;21(4):e0333505. doi: 10.1371/journal.pone.0333505 (PMC13075678; doi:10.1371/journal.pone.0333505)
Supplement: S1 File — S1 Table. Additional information on the Wheat Association Mapping Initiative (WAMI) population. This population was introduced by the International Maize and Wheat Improvement Center (CIMMYT) in 2009 and comprises 287 advanced elite lines. Based on the recommendations of CIMMYT researchers and experts, a sub-population of 170 lines was selected to ensure the maintenance of the desired level of genetic diversity. This sub-population is labeled as group S170. Among these 170 lines, those highlighted in green were utilized for the heat stress study, along with three Iranian cultivars listed at the end of the table. The genotypes were used with their respective names, which are provided in the first column. The pedigree information and selection history of the genotypes are presented in the columns labeled “Cross” and “Selection History,” respectively. S2 Table. Values of stress tolerance and sensitivity indices for all studied genotypes. The genotypes included in this study comprise a sub-population of the Wheat Association Mapping Initiative (WAMI), consisting of 153 advanced elite lines along with three Iranian cultivars used as controls (Pishtaz = 299, Qhods = 300, and Roshan = 301). The Gen column lists the genotypes, with their pedigree information provided in S1 Table of this supplementary material. S3 Table. Clustering of genotypes from the WAMI sub-population and control cultivars (additional genotype information is provided in S1 Table) into three groups: tolerant, semi-tolerant, and susceptible. Genotypes with HSI values < 0.8 were classified as tolerant, those with values between 0.8 and 1.2 as semi-tolerant, and those with values > 1.2 as susceptible genotypes. In several studies, other researchers have also utilized the HSI index to identify tolerant genotypes, and the results of our study align with their findings. S4 Table. Analysis of variance (ANOVA) for agronomic and quality-related traits in the Wheat Association Mapping Initiative (WAMI) panel (153 [file pone.0333505.s001.docx]

| **S1 Table.** Additional information on the Wheat Association Mapping Initiative (WAMI) population. This population was introduced by the International Maize and Wheat Improvement Center (CIMMYT) in 2009 and comprises 287 advanced elite lines (Lopes et al. 2015). Based on the recommendations of CIMMYT researchers and experts, a sub-population of 170 lines was selected to ensure the maintenance of the desired level of genetic diversity (Khodaee et al. 2021; Lopes et al. 2015; Sukumaran et al. 2015). This sub-population is labeled as group **S170**. Among these 170 lines, those highlighted in green were utilized for the heat stress study, along with three Iranian cultivars listed at the end of the table. The genotypes were used with their respective names, which are provided in the first column. The pedigree information and selection history of the genotypes are presented in the columns labeled "Cross" and "Selection History," respectively. | | | | | | | |
| --- | --- | --- | --- | --- | --- | --- | --- |
| Name | CIMMYT Code | GID | S170 | Cross | | Selection History | |
| 1 | 255 | 5E+06 | S170 | CNO79//PF70354/MUS/3/PASTOR/4/CROC_1/AE.SQUARROSA (224)//OPATA | | CMSS97M02939T-040Y-020Y-030M-040SY-020M-19Y-010M-0Y-0SY | |
| 2 | 218 | 4E+06 |  | ATTILA*2/4/CAR//KAL/BB/3/NAC | | CGSS96B00128F-099M-028Y-099M-28Y-0B-0SY | |
| 3 | 026 | 3E+05 | S170 | KAUZ*2//DOVE/BUC/3/KAUZ | | CRG802-6Y-010M-0Y-0HTY | |
| 4 | 116 | 5E+06 | S170 | SUNSU/CHIBIA | | CMSS00M02326S-030M-030WGY-030M-6M-0Y | |
| 5 | 039 | 4E+05 | S170 | ATTILA//ALTAR 84/AOS/3/ATTILA | | CMBW90M4859-0TOPY-8M-2Y-010M-010Y-9M-015Y-0Y | |
| 6 | 074 | 4E+06 |  | HUITES/4/CS/TH.SC//3*PVN/3/MIRLO/BUC | | CMSS94Y00476S-0300M-0100Y-0100M-4Y-8M-0Y-0HTY | |
| 7 | 024 | 3E+05 |  | FCT/3/GOV/AZ//MUS/4/DOVE/BUC | | CM99727-0M-14Y-0L-3Y-2Y-0Y-0HTY | |
| 8 | 222 | 4E+06 | S170 | ATTILA*2/PASTOR | | CGSS97Y00043F-099TOPB-055Y-099M-099Y-099M-12Y-0B | |
| 9 | 080 | 4E+06 | S170 | SW89.5181/KAUZ | | CMSS93B00824S-24Y-010M-010Y-010M-9Y-0M-0HTY | |
| 10 | 086 | 4E+06 |  | HUAYTU CIAT | | -0BOL | |
| 11 | 179 | 8E+05 | S170 | VEE#5/SARA//DUCULA | | CMBW89Y2411-16Y-010M-010Y-010M-0M-0SY-32Y-0B-0SY | |
| 12 | 162 | 2E+06 | S170 | HIDHAB | | -0DZA | |
| 13 | 139 | 3E+05 | S170 | ATTILA | | CM85836-50Y-0M-0Y-3M-0Y-0SY | |
| 14 | 156 | 3E+05 |  | ALTAR 84/AEGILOPS SQUARROSA (TAUS)//OPATA | | CMBW89Y3514-4Y-010M-010Y-62M-5Y-0M-0SY | |
| 15 | 078 | 2E+06 |  | OASIS/SKAUZ//4*BCN | | CMSS93Y04053M-3M-0Y-0HTY | |
| 16 | 094 | 4E+06 |  | SKAUZ*2/FCT | | CMBW91M02703F-0TOPY-24M-010Y-010M-010Y-1Y-0M-41Y-0B | |
| 17 | 240 | 5E+06 | S170 | KABY/BAV92/3/CROC_1/AE.SQUARROSA (224)//OPATA | | CMSS97M02975T-040Y-020Y-030M-040SY-020M-28Y-0M-0SY | |
| 18 | 051 | 3E+05 |  | URES/RAYON | | CMBW89M56-21M-010Y-010M-010M-010Y-9M-015Y-0Y | |
| 19 | 017 | 2E+05 | S170 | CAR422/ANA//URES | | CM93553-14M-0Y-0M-1Y-0B | |
| 20 | 195 | 4E+06 | S170 | CROC_1/AE.SQUARROSA (205)//KAUZ/3/ENEIDA | | CMSS95Y00148S-18Y-010M-050SY-010M-9SY-0Y-0SY | |
| 21 | 261 | 5E+06 | S170 | ALTAR 84/AEGILOPS SQUARROSA (TAUS)//OPATA/3/ATTILA | | CMSS99M00936S-0P0M-040SY-040M-040SY-16M-0ZTB-0SY | |
| 22 | 245 | 5E+06 | S170 | FRAME/BUCHIN | | CMSS97Y04123S-5Y-010M-010SY-010M-8SY-010M-0Y-0SY | |
| 23 | 033 | 3E+05 | S170 | TURACO/CHIL | | CM92354-57Y-0H-0SY-2M-0RES-0HTY | |
| 24 | 109 | 4E+06 | S170 | PBW450 | | -0NPL | |
| 25 | 274 | 6E+06 | S170 | WHEAR/KUKUNA/3/C80.1/3*BATAVIA//2*WBLL1 | | CGSS03B00080T-099Y-099M-099Y-099M-7WGY-0B | |
| 26 | 187 | 2E+06 |  | KAUZ/5/PAT10/ALD//PAT72300/3/PVN/4/BOW | | CMSS93B01334S-70Y-010M-010SY-010M-2SY-0M-0SY | |
| 27 | 228 | 4E+06 | S170 | ALTAR 84/AE.SQUARROSA (221)//PASTOR/3/PASTOR | | CMSS97Y06183T-040M-1Y-010M-010SY-010M-7SY-010M-0Y | |
| 28 | 015 | 80662 |  | PAT10/ALD//PAT72300/3/PVN/4/URES/5/PFAU | | CM87688-029TOPM-5Y-0H-0SY-3M-0Y | |
| 29 | 095 | 4E+06 |  | CNDO/R143//ENTE/MEXI_2/3/AEGILOPS SQUARROSA (TAUS)/4/WEAVER/5/2*KAUZ | | CMSS93B01824M-040Y-73Y-010M-010Y-010M-1Y-0M-0KBY | |
| 30 | 088 | 4E+06 | S170 | TARACHI F 2000 | | CRG2753.1-0B-099Y-099M-28Y-0B-0MEX | |
| 31 | 248 | 5E+06 |  | CROC_1/AE.SQUARROSA (224)//OPATA/3/PASTOR/4/PASTOR*2/OPATA | | CMSS98Y03432T-040M-0100M-040Y-020M-040SY-23M-0Y-0SY | |
| 32 | 254 | 5E+06 | S170 | KABY//2*ALUBUC/BAYA | | CMSS97M02966M-040Y-020Y-030M-040SY-020M-29Y-010M-0Y-0SY | |
| 33 | 281 | 6E+06 | S170 | PRL/2*PASTOR/4/CHOIX/STAR/3/HE1/3*CNO79//2*SERI | | CMSS02Y00596S-15Y-0M-099Y-1M-0WGY-0B | |
| 34 | 278 | 6E+06 | S170 | WHEAR//2*PRL/2*PASTOR | | CGSS03B00090T-099Y-099M-099Y-099M-17WGY-0B | |
| 35 | 279 | 6E+06 |  | WHEAR//2*PRL/2*PASTOR | | CGSS03B00090T-099Y-099M-099Y-099M-47WGY-0B | |
| 36 | 004 | 3895 | S170 | SERI M 82 | | CM33027-F-15M-500Y-0M-87B-0Y-0MEX | |
| 37 | 286 | 6E+06 |  | HEILO//MILAN/MUNIA | | CMSS02Y01475S-23Y-0M-099Y-3M-0WGY-0B | |
| 38 | 193 | 2E+06 |  | PASTOR//SITE/MO/3/CHEN/AEGILOPS SQUARROSA (TAUS)//BCN | | CMSS94Y02392T-030Y-0300M-0100Y-0100M-050SY-14M-0SY-0SY | |
| 39 | 020 | 1E+05 |  | PAT10/ALD//PAT72300/3/PVN/4/BOW | | CM84490-6M-0SY-0H-9Y-0M-5M-0HER | |
| 40 | 230 | 5E+06 | S170 | FLORKWA-1/DHARWAR DRY | | CMSS96M03160S-050M-050SY-040SY-030M-19SY-010M-0Y-0SY | |
| 41 | 055 | 4E+05 | S170 | HP 1731 | | -(RAJLAXMIN)-0IND | |
| 42 | 153 | 3E+05 | S170 | PARA2//JUP/BJY/3/VEERY#5.4/JUN/4/TUI | | CM107592-9Y-020Y-010M-6Y-010M-3Y-0M-0SY | |
| 43 | 219 | 3E+06 |  | KAMBARA2 | | CGSS96Y00151T-099B-099Y-099B-29Y-0B-0SY | |
| 44 | 098 | 5E+06 | S170 | KAUZ*2/TRAP//KAUZ/3/PASTOR/4/SKAUZ*2/SRMA | | CMSS97M04053T-040Y-040M-020Y-030M-015Y-24M-2Y-1M-0Y | |
| 45 | 273 | 6E+06 | S170 | WHEAR/4/SNI/TRAP#1/3/KAUZ*2/TRAP//KAUZ/5/C80.1/3*BATAVIA//2*WBLL1 | | CGSS03B00078T-099Y-099M-099Y-099M-16WGY-0B | |
| 46 | 290 | 5E+06 |  | FRET2*2/BRAMBLING | | CGSS01B00060T-099Y-099M-099M-099Y-099M-44Y-0B | |
| 47 | 266 | 6E+06 |  | ND643//2*PRL/2*PASTOR | | CGSS02B00112T-099B-099Y-099M-099Y-099M-1WGY-0B | |
| 48 | 130 | 88710 |  | URES/JUN//KAUZ | | CM96818-I-0Y-0M-0Y-5M-0RES | |
| 49 | 232 | 5E+06 |  | VEBOW/IRENA | | CMSS96M03267S-050M-050SY-040SY-030M-17SY-010M-0Y-0SY | |
| 50 | 064 | 4E+06 | S170 | CHUM18/5*BCN | | CMSS96Y03533M-0100M-4Y-0M-0HTY | |
| 51 | 174 | 2E+05 | S170 | KAUZ*2/BOW//KAUZ | | CRG905-13Y-010M-0Y | |
| 52 | 180 | 1E+06 | S170 | CLC89//ESDA/KAUZ/3/BJY/COC//PRL/BOW | | CMSS92Y02540T-29Y-015M-010SY-010SY-4M-0SY-0SY | |
| 53 | 066 | 4E+06 | S170 | MILAN/3/JUP/BJY//URES | | CMSS93B01074S-112Y-010M-010Y-010M-2Y-0M-0HTY | |
| 54 | 235 | 4E+06 | S170 | PASTOR//HXL7573/2*BAU | | CMSS97M00306S-0P5M-0P5Y-040Y-20M-0Y-0SY | |
| 55 | 183 | 1E+06 | S170 | TODY/3/JUP/BJY//SARA/4/TRAP#1/BOW/5/NL456/VEE#5 | | CMSS92M03451T-015M-0Y-0Y-050M-22Y-1M-0Y-0SY | |
| 56 | 263 | 4E+06 | S170 | PASTOR//TRAP#1/BOW/3/CHEN/AEGILOPS SQUARROSA (TAUS)//BCN | | CMSS94Y02321T-030Y-0300M-0100Y-0100M-9Y-6M-0Y-3PZ-0Y | |
| 57 | 021 | 42423 | S170 | VORONA/CNO79 | | CM76688-9Y-03M-02Y-2B-0Y | |
| 58 | 259 | 5E+06 | S170 | CHEN/AE.SQ//2*OPATA/3/BAV92/4/JARU | | CMSS99Y03521T-040M-040Y-040M-040SY-040M-4Y-010M-0ZTB-0SY | |
| 59 | 221 | 4E+06 |  | PBW65/2*PASTOR | | CGSS97Y00036M-099TOPB-067Y-099M-099Y-099B-16Y-0B | |
| 60 | 073 | 4E+06 |  | CHIR1//SHA5/WEAVER | | CMSS96Y04261S-040Y-020M-040Y-020Y-20M-0Y-0HTY | |
| 61 | 111 | 5E+06 | S170 | ATTILA/3*BCN*2//BAV92 | | CMSS97M04170F-040Y-040M-020Y-030M-015Y-7M-2Y-3M-0Y | |
| 62 | 285 | 6E+06 |  | ELVIRA/5/CNDO/R143//ENTE/MEXI75/3/AE.SQ/4/2*OCI/6/VEE/PJN//KAUZ/3/PASTOR | | CMSS02M01105T-030M-18Y-0M-099Y-2M-0WGY-0B | |
| 63 | 206 | 3E+06 | S170 | WEEBILL4 | | CGSS96Y00150T-099B-099Y-099B-23Y-0B-0SY | |
| 64 | 046 | 1E+06 | S170 | SERI/7C | | CMSS92Y02905S-17TLA-0TLA-0TLA-0TLA-1B-0HTY | |
| 65 | 212 | 4E+06 | S170 | URES/PRL//BAV92 | | CMSS95Y02695S-0100Y-0200M-050SY-050M-43SY-0Y-0SY | |
| 66 | 169 | 3E+05 | S170 | HXL-F86/2*BAU | | CMBW91Y03612M-030TOPM-4Y-010M-010Y-015M-8Y-0M-0SY | |
| 67 | 027 | 1E+06 |  | KAUZ*2/TRAP//KAUZ | | CRG744-9Y-010M-0Y-0HTY | |
| 68 | 034 | 1E+06 |  | KAUZ*3//TC*6/RL5406(RL6043) | | CRG1046-6Y-010M-0Y-0HTY-0B | |
| 69 | 029 | 3E+05 | S170 | PARA2//JUP/BJY/3/VEE/JUN/4/2*KAUZ | | CMBW89M7300-0TOPY-030M-8Y-010M-3Y-0M-1KBY-0M | |
| 70 | 090 | 4E+06 | S170 | SKAUZ*2/FCT | | CMBW91M02703F-0TOPY-24M-010Y-010M-010Y-1Y-0M-0HTY | |
| 71 | 271 | 6E+06 | S170 | CHEWINK | | CGSS03B00074T-099Y-099M-099Y-099M-2WGY-0B | |
| 72 | 155 | 3E+05 | S170 | URES/BBL//KAUZ/3/KAUZ | | CMBW89Y00770-0TOPM-7Y-010M-010SY-010M-0M-0SY | |
| 73 | 133 | 42274 | S170 | SITTA | | CM77091-14Y-04M-06Y-3B-1Y-0B | |
| 74 | 123 | 88208 | S170 | VEE#8/5/VEE/4/KLTO//S12/J9281.67/3/MO/JUP | | CM79922-11Y-025H-0SY-4M-0Y | |
| 75 | 104 | 5E+06 | S170 | BAV92/3/OASIS/SKAUZ//4*BCN/4/PASTOR | | CMSS98Y01925T-040M-8SY-0M-010Y-010M-3Y-3M-0Y | |
| 76 | 087 | 4E+06 | S170 | OTUS | | CMBW90Y3180-0TOPM-3Y-010M-010M-010Y-10M-015Y-0Y-1KBY-0KBY-0M-0HTY | |
| 77 | 083 | 4E+06 |  | CHEN/AEGILOPS SQUARROSA (TAUS)//BCN/3/2*KAUZ | | CMSS93B01856M-040Y-43Y-010M-010Y-010M-1Y-0M-2KBY-0KBY-0M-0HTY | |
| 78 | 107 | 5E+06 |  | TACUPETO F2001*2/KUKUNA | | CGSS00B00169T-099TOPY-099M-099Y-099M-9CEL-0B | |
| 79 | 293 | 5E+06 | S170 | CROC_1/AE.SQUARROSA (205)//BORL95/3/PRL/SARA//TSI/VEE#5/4/FRET2 | | CMSA00Y00817T-040M-0P0Y-040M-040SY-030M-10ZTM-0ZTY-0M-0SY | |
| 80 | 118 | 5E+06 | S170 | WBLL1*2/VIVITSI | | CGSS01Y00058T-099M-099Y-099M-099M-23Y-0B | |
| 81 | 012 | 85599 | S170 | CHOIX M 95 | | CM90722-22Y-0M-0Y-5M-0Y-0MEX | |
| 82 | 265 | 6E+06 | S170 | ND643/2*WAXWING | | CGSS02B00107T-099B-099Y-099M-099Y-099M-11WGY-0B | |
| 83 | 142 | 85587 | S170 | PRINIA | | CM90722-22Y-0M-0Y-3M-0Y | |
| 84 | 252 | 5E+06 | S170 | ALTAR 84/AE.SQ//2*OPATA/3/PIFED | | CMSS98Y00581S-0100M-040Y-020M-040SY-29M-0Y-0SY | |
| 85 | 057 | 2E+06 | S170 | KAUZ/RAYON | | CRG2756.1-0B-099Y-099M-24Y-0B-0HTY | |
| 86 | 236 | 5E+06 |  | SRMA/TUI//PASTOR | | CMSS97M00386S-040M-040SY-030M-040SY-33M-0Y-0SY | |
| 87 | 264 | 4E+06 | S170 | PRL/2*PASTOR | | CGSS97Y00034M-099TOPB-027Y-099M-099Y-099M-27Y-0B | |
| 88 | 178 | 8E+05 | S170 | TUI/3/TMP64/TWN//SDY/4/RAYON | | CMBW89Y01466-0TOPM-6Y-010M-010Y-010M-0M-0SY-12Y-0B-0SY | |
| 89 | 191 | 1E+06 |  | JUP/BJY//URES/3/HD2206/HORK//BUC/BUL | | CMSS92M00061S-015M-0Y-0Y-050M-4Y-2M-0Y-0SY | |
| 90 | 152 | 3E+05 | S170 | VEE/PJN//TUI | | CM107563-14Y-020Y-010M-1Y-010M-0Y-0SY | |
| 91 | 234 | 5E+06 |  | BJY/COC//PRL/BOW/3/FRTL | | CMSS96M03436S-050M-050SY-040SY-030M-29SY-010M-0Y-0SY | |
| 92 | 054 | 5E+05 | S170 | TIA.2/KAUZ | | CM103733-23M-030Y-020Y-010M-5Y-010Y-0M-0HTY | |
| 93 | 035 | 1E+06 | S170 | KAUZ*2//TC*6/RL6081/3/KAUZ | | CRG1090-3Y-010M-0Y-0HTY-0B | |
| 94 | 229 | 5E+06 |  | MILAN/KAUZ//PASTOR | | CMSS96M03124S-050M-050SY-040SY-030M-25SY-010M-0Y-0SY | |
| 95 | 044 | 1E+06 | S170 | MNCH/3*BCN | | CMBW90Y5756-0TOPM-14Y-010M-010M-010Y-5M-015Y-0Y | |
| 96 | Pishtaz11 |  |  |  | |  | |
| 97 | Sirvan10 |  |  |  | |  | |
| 98 | 170 | 2E+06 | S170 | VI/PIFED//VEE#8 | | A9071-2T-1M-1B-2T-0T-0ARG | |
| 99 | 239 | 5E+06 |  | MILAN/KAUZ/3/URES/JUN//KAUZ/4/CROC_1/AE.SQUARROSA (224)//OPATA | | CMSS97M02956T-040Y-040M-040SY-030M-040SY-13M-0Y-0SY | |
| 100 | 138 | 80512 | S170 | F60314.76/MRL//CNO79 | | CM77694-A-1Y-02M-05Y-1B-1Y-0B | |
| 101 | 184 | 1E+06 | S170 | CROC_1/AE.SQUARROSA (205)//JUP/BJY/3/SKAUZ/4/KAUZ | | CMSS92M03104T-015M-0Y-0Y-050M-26Y-2M-0Y-0SY | |
| 102 | 243 | 5E+06 |  | BJY/COC//PRL/BOW/3/MILAN/KAUZ/4/BAV92 | | CMSS97M03147T-040Y-030M-040SY-030M-040SY-1M-0Y-0SY | |
| 103 | 009 | 6E+05 | S170 | MYNA/VUL//PRL | | CM97958-0M-7Y-030M-030M-3Y-0Y | |
| 104 | 049 | 3E+05 | S170 | TILHI | | CMBW90M4860-0TOPY-16M-1Y-010M-010Y-1M-015Y-0Y | |
| 105 | 047 | 4E+05 |  | SERI/NKT//2*KAUZ | | CMBW90Y3411-0TOPM-2Y-010M-010M-010Y-8M-015Y-0Y | |
| 106 | 067 | 3E+06 | S170 | NL 750 | | -0NPL | |
| 107 | 093 | 1E+06 |  | INQALAB 91 | | PB19545-9A-0A-0PAK | |
| 108 | 063 | 4E+06 | S170 | BUC/PRL//WEAVER | | CMSS92M00233S-015M-0Y-0Y-050M-26Y-3M-0Y-0HTY | |
| 109 | 217 | 4E+06 |  | NAI60/HEINE VII//BUC/3/PSN/BOW//TUI | | CMSW95WM00031S-0100M-050SY-050M-050SY-030M-7SY-0M-0SY | |
| 110 | 126 | 42174 | S170 | CULIACAN T 89 | | CM74849-2M-2Y-3M-2Y-0B-46M-0Y-0MEX | |
| 111 | 132 | 1E+05 |  | HI.1077 | |  | |
| 112 | 140 | 2E+05 |  | PFAU/VEE#9 | | CM88703-9Y-0H-0Y-4M-0Y-0SY | |
| 113 | 096 | 4E+06 |  | KETUPA*2/PASTOR | | CMSS96M05146F-040Y-050M-040Y-0100M-020Y-13M-0Y-1M-0Y | |
| 114 | 163 | 35054 | S170 | W3918A/JUP | | CM40096-8M-7T-0M | |
| 115 | 001 | 5E+05 | S170 | COOK/VEE//DOVE/SERI/3/BJY/COC | | CM90507-20Y-0M-0Y-1M-0Y | |
| 116 | 014 | 3E+05 | S170 | KAUZ*2/MNV//KAUZ | | CRG958-10Y-010M-0Y | |
| 117 | 277 | 6E+06 |  | WHEAR/KURUKU/3/C80.1/3*BATAVIA//2*WBLL1 | | CGSS03B00085T-099Y-099M-099Y-099M-27WGY-0B | |
| 118 | 136 | 82710 | S170 | TIA.1 | | CIGM81.51-26B-2Y-1B-2Y-1B-4Y-1B-0Y | |
| 119 | 084 | 4E+06 |  | CHEN/AEGILOPS SQUARROSA (TAUS)//BCN/3/KAUZ | | CMSS93Y00868S-13Y-3KBY-010M-010Y-1M-0KBY-0M-10KBY-0Y-0HTY | |
| 120 | 238 | 5E+06 |  | CNO79//PF70354/MUS/3/PASTOR/4/BAV92 | | CMSS97M02936T-040Y-030M-040SY-030M-040SY-22M-0Y-0SY | |
| 121 | 037 | 4E+05 | S170 | PRL/VEE#6 | | CM64624-2Y-1M-4Y-0M-19Y-0M-0HTY-0B | |
| 122 | 099 | 5E+06 | S170 | REH/HARE//2*BCN/3/CROC_1/AE.SQUARROSA (213)//PGO/4/HUITES | | CMSS97M04104T-040Y-020Y-030M-020Y-040M-1Y-2M-0Y | |
| 123 | 150 |  |  | JUPARE C 2001 | | CD91Y636-1Y-040M-030Y-1M-0Y-0B-1Y-0B-0MEX | |
| 124 | 269 | 6E+06 | S170 | SAAR/WBLL1 | | CGSS03B00166S-099M-099Y-099M-1WGY-0B | |
| 125 | 214 | 4E+06 | S170 | SOROCA | | CMSS96Y02567S-040Y-020M-050SY-020SY-35M-0Y | |
| 126 | 059 | 2E+06 | S170 | PASTOR/2*SITTA | | CMSS92Y01650T-41Y-010M-010Y-010Y-2M-0Y-0HTY | |
| 127 | 168 | 3E+05 | S170 | CNDO/R143//ENTE/MEXI_2/3/AEGILOPS SQUARROSA (TAUS)/4/WEAVER | | CMBW89Y3538-9Y-010M-010Y-28M-1Y-0M-1KBY-0M-0SY | |
| 128 | vee/nac13 |  |  |  | |  | |
| 129 | 224 | 4E+06 |  | PASTOR//HXL7573/2*BAU | | CMSS97M00306S-0P20M-0P20Y-97M-010Y | |
| 130 | 062 | 4E+06 | S170 | BOW/PRL*3/6/WRM/4/FN/3*TH//K58/2*N/3/AUS-6869/5/PELOTAS-ARTHUR/7/HE1/3*CNO79//2*SERI | | CMSS95Y01545S-0100Y-51DH-0B-0HTY | |
| 131 | 275 | 6E+06 | S170 | WHEAR/JARU/3/C80.1/3*BATAVIA//2*WBLL1 | | CGSS03B00083T-099Y-099M-099Y-099M-10WGY-0B | |
| 132 | 231 | 5E+06 |  | CNDO/R143//ENTE/MEXI_2/3/AEGILOPS SQUARROSA (TAUS)/4/WEAVER/5/PASTOR | | CMSS96M03230S-050M-050SY-040SY-030M-19SY-010M-0Y-0SY | |
| 133 | 200 |  |  | JUPARE C 2001 | | CD91Y636-1Y-040M-030Y-1M-0Y-0B-1Y-0B-0MEX | |
| 134 | Aflak15 |  |  |  | |  | |
| 135 | 267 | 6E+06 |  | KIRITATI//2*PRL/2*PASTOR | | CGSS02B00121T-099B-099Y-099M-099Y-099M-7WGY-0B | |
| 136 | 016 | 1E+05 | S170 | PRINIA | | CM90722-17Y-0H-0Y-5M-0Y-1M-0HER | |
| 137 | Sepahan12 |  |  |  | |  | |
| 138 | 146 | 5E+06 |  | KITE/GLEN | | CM90734-3Y-0M-0Y-4M-0Y-4M-0Y-4M-1J-0J-0ARG | |
| 139 | 105 | 5E+06 | S170 | CROC_1/AE.SQUARROSA (205)//KAUZ/3/2*KAUZ*2/YACO//KAUZ | | CMSS98Y02488M-040M-0100M-040Y-040M-030Y-30M-1Y-0M | |
| 140 | 006 | 6E+05 | S170 | KAUZ/GEN | | CRG178.2-65B-0Y-030M-3Y-3Y-0M | |
| 141 | 241 | 5E+06 |  | BOW//BUC/BUL/3/KAUZ/4/BAV92/5/MILAN/KAUZ | | CMSS97M03030T-040Y-020Y-030M-040SY-020M-11Y-0M-0SY | |
| 142 | 134 | 6E+05 |  | URES//BUC/FLK/3/KAUZ | | CM96817-C-0Y-0M-0Y-4M-0RES-0SY | |
| 143 | 204 | 3E+06 | S170 | PEWIT1 | | CGSS96Y00138T-099B-099Y-099B-21Y-0B-0SY | |
| 144 | 197 | 4E+06 |  | PASTOR/BAV92 | | CMSS96M03183S-78M-010SY-0M-0Y-0SY | |
| 145 | 276 | 6E+06 | S170 | WHEAR/TUKURU/3/C80.1/3*BATAVIA//2*WBLL1 | | CGSS03B00084T-099Y-099M-099Y-099M-5WGY-0B | |
| 146 | 176 | 8E+05 | S170 | CAR853/COC//VEE/3/BOW/4/TUI/5/TUI | | CMBW89Y00641-0TOPM-26Y-010M-010Y-010M-0M-0SY-9Y-0B-0SY | |
| 147 | 203 | 3E+06 | S170 | BABAX.1B.1B*3/PRL | | CGSS96Y00136M-099B-099Y-099B-1Y-0B-0SY | |
| 148 | 151 | 3E+05 | S170 | PJN/BOW//OPATA | | CM107553-2Y-020Y-010M-1Y-010M-2Y-0M-0SY | |
| 149 | 003 | 41868 |  | PFAU/VEE#5 | | CM84191-4M-0Y-0M-4Y-0M | |
| 150 | 256 | 5E+06 | S170 | BUC/MN72253//PASTOR/3/BAV92 | | CMSS97M03203T-040Y-020Y-030M-040SY-020M-17Y-010M-0Y-0SY | |
| 151 | 131 | 72533 | S170 | PROINTA FEDERAL | | CM33203-M-8M-8Y-1M-1Y-1M-0Y-1T-2T-0ARG | |
| 152 | 141 | 2E+05 | S170 | BAU/OPATA | | CM90555-33M-0Y-0M-3Y-0B-0SY | |
| 153 | 246 | 5E+06 |  | TEMPORALERA M 87*2/KONK | | CGSS99B00030F-099Y-099M-099Y-099M-45Y-0B | |
| 154 | 171 | 4E+05 |  | GOV/AZ//MUS/3/SARA | | CM80783-4Y-025H-0SY-3M-3Y-0M-0SY | |
| 155 | 237 | 5E+06 |  | SKAUZ/PASTOR/3/CROC_1/AE.SQUARROSA (224)//OPATA | | CMSS97M02925T-040Y-040M-040SY-030M-040SY-9M-0Y-0SY | |
| 156 | 192 | 1E+06 | S170 | SITE/PIOS | | CMSS92M01395S-015M-0Y-0Y-050M-1Y-3M-0Y-0SY | |
| 157 | 068 | 4E+05 |  | PUNJAB 96 | | -0PAK | |
| 158 | 258 | 5E+06 |  | CROC_1/AE.SQUARROSA (224)//OPATA/3/BJY/COC//PRL/BOW/4/BJY/COC//PRL/BOW | | CMSS99Y03513T-040M-040Y-040M-040SY-040M-18Y-010M-0ZTB-0SY | |
| 159 | 250 |  |  | JUPARE C 2001 | | CD91Y636-1Y-040M-030Y-1M-0Y-0B-1Y-0B-0MEX | |
| 160 | 284 | 6E+06 | S170 | PRL/SARA//TSI/VEE#5/3/TILHI/4/ATTILA/2*PASTOR | | CMSS02Y02176T-060M-25Y-0M-099Y-4M-0WGY-0B | |
| 161 | 089 | 4E+06 | S170 | SITE/MO/4/NAC/TH.AC//3*PVN/3/MIRLO/BUC | | CMSS93B00567S-72Y-010M-010Y-010M-9Y-0M-0HTY | |
| 162 | 182 | 1E+06 | S170 | 3VASKAR/G303.1M.1.3.2.2.2//KAUZ/3/SKAUZ/4/KAUZ | | CMSS92M03140T-015M-0Y-0Y-050M-7Y-1M-0Y-0SY | |
| 163 | 028 | 3E+05 |  | LIRA/BUC | | CM93672-13M-0Y-0M-7Y-0B-0HTY | |
| 164 | 205 | 2E+06 | S170 | FRET2 | | CGSS96Y00146T-099B-099Y-099B-12Y-0B | |
| 165 | 244 | 5E+06 |  | KAUZ/BAV92/3/BJY/COC//PRL/BOW | | CMSS97M03271T-040Y-030M-040SY-030M-040SY-12M-0Y-0SY | |
| 166 | 040 | 1E+06 | S170 | FANG60/7C | | CMSS92Y02902S-4TLA-0TLA-0TLA-0TLA-2B-0HTY | |
| 167 | 106 | 5E+06 | S170 | WBLL1*2/KUKUNA | | CGSS00B00160T-099TOPY-099M-099Y-099M-7CEL-0B | |
| 168 | 007 | 80836 |  | KEA/TOW//LIRA | | CM90450-1Y-0M-0Y-3M-0Y | |
| 169 | 117 | 5E+06 | S170 | WBLL1*2/4/YACO/PBW65/3/KAUZ*2/TRAP//KAUZ | | CGSS01Y00054T-099M-099Y-099M-099M-20Y-0B | |
| 170 | 045 | 16004 | S170 | RAYON F 89 | | CM90315-A-2B-2Y-1B-0Y-0MEX | |
| 171 | 032 | 5E+05 | S170 | TRAP#1/BOW | | CM84548-34Y-0M-0Y-8M-0Y-0HTY | |
| 172 | 023 | 16122 | S170 | BACANORA T 88 | | CM67458-4Y-1M-3Y-1M-5Y-0B-0MEX | |
| 173 | 061 | 3E+06 | S170 | BL 1724 | | -0NPL | |
| 174 | 048 | 3E+05 | S170 | STAR//KAUZ/STAR | | CMBW90M4968-0TOPY-095M-095Y-095M-2Y-0M | |
| 175 | 108 | 4E+06 |  | HD2687 | | -0IND | |
| 176 | 177 | 8E+05 | S170 | NANJING 8646/KAUZ//BCN | | CMBW89Y00966-0TOPM-19Y-010M-010Y-010M-0M-0SY-35Y-0B-0SY | |
| 177 | 282 | 6E+06 | S170 | PRL/2*PASTOR/4/CHOIX/STAR/3/HE1/3*CNO79//2*SERI | | CMSS02Y00596S-43Y-0M-099Y-2M-0WGY-0B | |
| 178 | 018 | 4248 |  | CIANO T 79 | | CM31678-R-4Y-2M-21Y-0M-0MEX | |
| 179 | 253 | 5E+06 | S170 | KRICHAUFF/2*PASTOR | | CMSS99Y05560T-2M-1Y-010M-010SY-3M-0Y-0SY | |
| 180 | 147 | 3E+05 |  | CHIL/BUC | | CM93687-23Y-0M-0Y-1M-0RES-0SY | |
| 181 | 158 | 9E+05 |  | SHUHA | | SWM11508-1AP-1AP-4AP-2AP-1AP-0AP | |
| 182 | 216 | 4E+06 | S170 | URES/JUN//KAUZ/3/BAV92 | | CMSS97M00339S-0P5M-0P5Y-79M-0Y | |
| 183 | 129 | 88701 | S170 | GIM/LIRA | | CM95981-75Y-0M-0Y-1M-0RES | |
| 184 | 056 | 1E+06 | S170 | IAS62/ALDAN//2*SKAUZ | | CMBW91M03458M-0TOPY-22M-2Y-010M-2KBY-1KBY-0M-0KBY-0HTY | |
| 185 | 292 | 5E+06 | S170 | CROC_1/AE.SQUARROSA (205)//BORL95/3/PRL/SARA//TSI/VEE#5/4/FRET2 | | CMSA00Y00817T-040M-0P0Y-040M-040SY-030M-1ZTM-0ZTY-0M-0SY | |
| 186 | 058 | 2E+06 | S170 | KEA/TAN/4/TSH/3/KAL/BB//TQFN/5/PAVON/6/SW89.3064 | | CMSS92Y01399T-2Y-010M-010Y-010Y-5M-0Y-0HTY | |
| 187 | 019 | 2E+05 | S170 | HD2206/HORK//BUC/BUL | | CM88500-25M-0Y-0M-10Y-0M | |
| 188 | 164 | 3E+05 |  | JUN/GEN | | CM85663-6Y-0H-0Y-1M-0Y | |
| 189 | 148 | 3E+05 |  | FILIN | | CM97334-0M-21Y-030M-2Y-3Y-0M-0SY | |
| 190 | 043 | 4E+05 |  | KAUZ//ALTAR 84/AOS | | CM111633-6M-020Y-010M-010Y-010M-2Y-0M | |
| 191 | 097 | 5E+06 |  | WEAVER/3/SAPI/TEAL//HUI/4/CROC_1/AE.SQUARROSA (213)//PGO/5/SKAUZ*2/SRMA | | CMSS97M03924T-040Y-020Y-030M-020Y-040M-5Y-1M-0Y | |
| 192 | 287 | 5E+06 |  | KAUZ//ALTAR 84/AOS/3/MILAN/KAUZ/4/HUITES | | CMSS97M03912T-040Y-020Y-030M-020Y-040M-4Y-3M-0Y | |
| 193 | 127 | 88509 |  | KITE/PGO | | CM90738-6Y-0M-0Y-4M-0Y | |
| 194 | 102 | 5E+06 | S170 | CROC_1/AE.SQUARROSA (205)//BORL95/3/PASTOR | | CMSS97M04895S-030M-020Y-030M-015Y-27M-2Y-3M-0Y | |
| 195 | 233 | 5E+06 | S170 | PASTOR/DHARWAR DRY | | CMSS96M03282S-050M-050SY-040SY-030M-1SY-010M-0Y-0SY | |
| 196 | 115 | 5E+06 | S170 | TUKURU//BAV92/RAYON | | CMSS00M01852S-030M-030WGY-030M-15M-0Y | |
| 197 | 167 | 3E+05 | S170 | VEE#8//JUP/BJY/3/F3.71/TRM/4/BCN/5/KAUZ | | CMBW89Y01242-0TOPM-29Y-010M-1Y-0M-0SY | |
| 198 | 025 |  |  | INIFAP M 97 | | CMBW89Y01231-0TOPM-16Y-010M-1Y-010M-5Y-0M-4KBY-0M | |
| 199 | 257 | 5E+06 | S170 | SCA/AE.SQUARROSA (409)//PASTOR/3/PASTOR | | CMSS99Y03439T-040M-040Y-040M-040SY-040M-6Y-010M-0ZTB-0SY | |
| 200 | 220 |  |  | PRL/2*PASTOR | | CGSS97Y00034M-099TOPB-027Y-099M-099Y-099M-27Y-0B | |
| 201 | 289 | 5E+06 | S170 | PAURAQUE | | CGSS01B00055T-099Y-099M-099M-099Y-099M-63Y-0B | |
| 202 | 100 |  |  | JUPARE C 2001 | | CD91Y636-1Y-040M-030Y-1M-0Y-0B-1Y-0B-0MEX | |
| 203 | 135 | 86005 | S170 | ARIVECHI M 92 | | CM100587-E-0M-0Y-030M-8Y-1Y-0M-0MEX | |
| 204 | 065 | 2E+06 |  | LAJ3302/2*MO88 | | CMSS92Y01621T-9Y-010M-010Y-010Y-4M-0Y-0HTY | |
| 205 | 202 | 3E+06 | S170 | KAMBARA1 | | CGSS95B00016F-099Y-099B-099Y-099B-20Y-0B-0SY | |
| 206 | 041 | 4E+05 |  | HP 1761 | | -(JAGUISH)-0IND | |
| 207 | 050 |  |  | JUPARE C 2001 | | CD91Y636-1Y-040M-030Y-1M-0Y-0B-1Y-0B-0MEX | |
| 208 | 291 | 5E+06 | S170 | BECARD | | CGSS01B00063T-099Y-099M-099M-099Y-099M-83Y-0B | |
| 209 | 077 | 4E+06 | S170 | MINO | | CMSS94Y02299T-030Y-0300M-0100Y-0100M-4Y-8M-0Y-0HTY | |
| 210 | 119 | 4E+05 |  | F6.74/BUN//SIS/3/YR/PAM | | CM88533-73M-0Y-0M-9Y-0M | |
| 211 | 213 | 4E+06 | S170 | VOROBEY | | CMSS96Y02555S-040Y-020M-050SY-020SY-27M-0Y | |
| 212 | 188 | 2E+06 | S170 | MRL/BUC//LIRA/5/BB//TOB/CNO67/3/HUAC/4/TI-R/3/BB/PL//SX | | CMSS93B01449S-32Y-010M-010SY-010M-10SY-0M-0SY | |
| 213 | 154 | 7E+05 |  | VEE/PJN//2*TUI | | CM112735-0TOPY-18M-020Y-010M-4Y-010M-6Y-0M-1KBY-0M | |
| 214 | 159 | 2E+06 | S170 | PROINTA GRANAR | | AMX14913-501P-1N-3B-1P-0P-0ARG | |
| 215 | 280 | 6E+06 | S170 | CNDO/R143//ENTE/MEXI_2/3/AEGILOPS SQUARROSA (TAUS)/4/WEAVER/5/2*KAUZ/6/PRL/2*PASTOR | | CMSS02Y00504S-11Y-0M-099Y-4M-0WGY-0B | |
| 216 | 165 | 3E+05 |  | K134(60)/VEE//BOW/PVN | | CM103578-6M-030Y-020Y-010M-3Y-010Y-0M-0SY | |
| 217 | 002 | 6E+05 |  | JUP/ZP//COC/3/PVN/4/GEN | | CM93697-17M-0Y-0M-4Y-0B | |
| 218 | 186 | 2E+06 |  | VEE#5//PF70354/MUS/3/PIFED/4/OR791432/VEE#3.2 | | CMSS93Y03695T-23Y-010SY-010M-010SY-10M-0Y-0SY | |
| 219 | 124 | 88522 |  | IRENA | | CM91575-7Y-0M-0Y-1M-0Y | |
| 220 | 103 | 5E+06 | S170 | CHEN/AE.SQ//WEAVER/3/SSERI1 | | CMSS98Y00278S-020Y-030M-020Y-040M-8Y-2M-0Y | |
| 221 | 144 | 4E+05 |  | HUITES F 95 | | CM67395-2M-3E-1E-1E-0E-0MEX-0SY | |
| 222 | 011 | 68315 | S170 | TIA.3 | | CIGM81.69-16B-4Y-3B-1Y-2B-4Y-5B-0Y | |
| 223 | 160 | 1E+06 |  | TZPP/SERI//BUC | | A9078.1T-1M-1B-2T-1T-0T-0ARG | |
| 224 | 110 | 5E+06 | S170 | MILAN/S87230//BAV92 | | CMSS97M03689T-040Y-030M-020Y-030M-015Y-30M-3Y-1M-0Y | |
| 225 | 185 | 1E+06 | S170 | PASTOR/3/VEE#5//DOVE/BUC | | CMSS93Y00302S-1Y-010Y-010M-010Y-4M-0Y-0SY | |
| 226 | 052 | 1E+06 | S170 | COMARA/TEG//WEAVER/3/LAJ3302 | | CMSS92Y01592T-10Y-010M-010Y-010Y-7M-0Y-0HTY | |
| 227 | 225 | 4E+06 |  | SOKOLL | | CMSS97M00316S-0P20M-0P20Y-51M-010Y | |
| 228 | 149 | 5E+05 | S170 | PRL/SARA//TSI/VEE#5 | | CM103448-41M-030Y-020Y-010M-2Y-010Y-0M-0SY | |
| 229 | 005 | 42893 | S170 | VORONA/GEN | | CM76689-23Y-05M-010Y-1B-3Y-0B | |
| 230 | 283 | 6E+06 | S170 | PFAU/MILAN/5/CHEN/AEGILOPS SQUARROSA (TAUS)//BCN/3/VEE#7/BOW/4/PASTOR | | CMSS02Y00613S-59Y-0M-099Y-5M-0WGY-0B | |
| 231 | 042 | 30709 |  | HYBRID DELHI 2172 | | -0IND | |
| 232 | 112 | 5E+06 | S170 | TOBA97/PASTOR | | CMSS97M05756S-040M-020Y-030M-015Y-3M-3Y-2M-0Y | |
| 233 | 113 | 5E+06 | S170 | FRET2*2/4/SNI/TRAP#1/3/KAUZ*2/TRAP//KAUZ | | CGSS00B00153T-099TOPY-099M-099Y-099M-20CEL-0B | |
| 234 | 211 | 2E+06 | S170 | PASTOR/3/MUNIA//CHEN/ALTAR 84/5/CNDO/R143//ENTE/MEXI_2/3/AEGILOPS SQUARROSA (TAUS)/4/WEAVER | | CMSS94Y02393T-030Y-0300M-0100Y-0100M-050SY-18M-0SY-0SY | |
| 235 | 013 | 2E+05 | S170 | KAUZ*2/FN//KAUZ | | CRG707-2Y-010M-0Y | |
| 236 | 008 | 6E+05 |  | LIRA/URES//MILO 9G19-2-26 | | CM99708-0M-1LMP-030M-030M-1Y-0M | |
| 237 | 053 | 1E+06 |  | PICUS/4/CS5A/5RL-1//BUC/BJY/3/ALD/PVN/5/LAJ3302 | | CMSS92Y01915T-7Y-010M-010Y-3KBY-1M-0Y-0HTY | |
| 238 | 072 | 1E+06 |  | CAZO/KAUZ//KAUZ | | CMBW90Y3284-0TOPM-14Y-010M-010M-010Y-6M-015Y-0Y-0HTY | |
| 239 | 199 | 3E+06 |  | MILVUS2 | | CGSS95B00010T-099Y-099B-099Y-099B-37Y-0B-0SY | |
| 240 | 120 | 41372 | S170 | PASTOR | | CM85295-0101Y-2M-0Y-0M-1Y-0M | |
| 241 | 038 | 2E+05 | S170 | UP 2338 | | -0IND | |
| 242 | 071 | 4E+06 | S170 | TIA.4/WL6572//RL6043/3*GEN/3/LUAN | | CMSS92M02814T-015M-0Y-0Y-050M-26Y-1M-0Y-0HTY | |
| 243 | 128 | 88442 |  | PSN/BOW//SERI | | CM92909-6M-0Y-0M-7Y-0B | |
| 244 | 189 | 2E+06 |  | SAAR | | CG25-099Y-099M-4Y-2M-2Y-0B-0SY | |
| 245 | 036 | 1E+06 |  | KAUZ*2/YACO//KAUZ | | CRG873-5Y-010M-0Y-0HTY-0B | |
| 246 | 210 | 4E+06 |  | SUJATA/SERI | | CMSS93Y02629S-91Y-010SY-010Y-015SY-1Y-05B-0Y-0SY | |
| 247 | 161 | 1E+06 | S170 | PIFED/DERN | | A11688-5T-0M-2B-1T-1T-0T-0ARG | |
| 248 | 201 | 3E+06 | S170 | WEEBILL1 | | CGSS95B00014T-099Y-099B-099Y-099B-41Y-0B-0SY | |
| 249 | 194 | 4E+06 | S170 | FILIN/IRENA/5/CNDO/R143//ENTE/MEXI_2/3/AEGILOPS SQUARROSA (TAUS)/4/WEAVER | | CMSS94Y02520T-030Y-0300M-0100Y-0100M-16Y-8M-0Y-0SY | |
| 250 | 196 | 4E+06 |  | F60314.76/MRL//CNO79/3/CHIL/PRL | | CMSS95Y02750S-0100Y-0200M-050SY-050M-46SY-0Y-0SY | |
| 251 | 137 | 85861 |  | RL6043/4*NAC | | CMH83.2551-A-2B-1Y-2B-0Y | |
| 252 | 122 | 2457 |  | PAVON | | CM8399 | |
| 253 | 175 | 2E+06 |  | SIMORGH | | CG29-099Y-099M-10Y-3M-1Y-0B-0SY | |
| 254 | 268 | 6E+06 |  | KIRITATI//2*ATTILA*2/PASTOR | | CGSS02B00130T-099B-099Y-099M-099Y-099M-10WGY-0B | |
| 255 | 227 | 5E+06 | S170 | MILAN/KAUZ//PRINIA/3/BAV92 | | CMSS97M02941T-040Y-020Y-030M-040Y-020M-1Y-0M | |
| 256 | 260 | 5E+06 |  | TIE CHUAN 1*2/3/HE1/3*CNO79//2*SERI | | CMSS99M01648F-040Y-040M-040SY-040M-040SY-15M-0ZTB-0SY | |
| 257 | 145 | 2E+05 |  | KEA/BUC//FCT | | CM85839-16Y-0M-0Y-4M-0Y-0SY | |
| 258 | Bahar14 |  |  |  | |  | |
| 259 | 121 | 14103 |  | GALVEZ S 87 | | CM33483-C-7M-1Y-0M-5B-0Y-0MEX | |
| 260 | 076 | 2E+06 | S170 | KAUZ/WEAVER | | CMSS93Y00076S-1DH-1B-0100B-0HTY | |
| 261 | 060 | 4E+05 |  | BHRIKUTI | | NL623-0NPL | |
| 262 | 031 | 3E+05 |  | TOBARITO M 97 | | CM103379-2M-030M-020Y-010M-2Y-010Y-0M | |
| 263 | 209 | 4E+06 | S170 | BABAX/KS93U76//BABAX | | CGSS96B00219S-099B-022Y-13B-0Y-0SY | |
| 264 | 082 | 4E+06 |  | GUAM92/KAUZ | | CMSS94B00008S-0300M-0100Y-0100M-18Y-7M-0Y-0HTY | |
| 265 | 251 | 5E+06 | S170 | CROC_1/AE.SQUARROSA (224)//OPATA/3/PASTOR/4/JARU | | CMSS98M00811T-040Y-0100M-040Y-020M-040SY-25M-0Y-0SY | |
| 266 | 247 | 5E+06 | S170 | FRAME*2/3/URES/JUN//KAUZ | | CMSS98Y03487F-040M-0100M-040Y-020M-040SY-13M-0Y-0SY | |
| 267 | 010 |  |  | JUPARE C 2001 | | CD91Y636-1Y-040M-030Y-1M-0Y-0B-1Y-0B-0MEX | |
| 268 | 070 | 4E+06 |  | SW89-5124*2/FASAN | | CMBW91Y03050F-030TOPM-2Y-010M-010Y-010M-6Y-0M-4PR-0B-1PR-0B-0HTY-0HTY | |
| 269 | 215 | 4E+06 |  | FILIN/2*PASTOR | | CMSS96Y03242M-050M-9Y-010M-010SY-010M-1SY-0M-0SY | |
| 270 | 173 | 1E+06 |  | TUI*2/MILAN | | CMBW90Y4025-0TOPM-18Y-010M-010M-010SY-3M-015SY-0SY | |
| 271 | 198 | 3E+06 | S170 | BARBET1 | | CGSS95B00006T-099Y-099B-099Y-099B-6Y-0B-0SY | |
| 272 | 091 | 2E+06 | S170 | SURUTU-CIAT | | -0BOL | |
| 273 | 114 | 5E+06 | S170 | ALTAR 84/AE.SQUARROSA (221)//3*BORL95/3/URES/JUN//KAUZ/4/WBLL1 | | CMSS00M00510T-040Y-030M-030WGY-030M-3M-0Y | |
| 274 | 226 | 4E+06 | S170 | CROC_1/AE.SQUARROSA (213)//PGO/3/BAV92 | | CMSS97M00814S-030M-040SY-010M-010SY-19Y-0M | |
| 275 | 207 | 4E+06 |  | ATTILA*2/9/KT/BAGE//FN/U/3/BZA/4/TRM/5/ALDAN/6/SERI/7/VEE#10/8/OPATA | | CGSS96B00119F-099M-061Y-099M-32Y-0B-0SY | |
| 276 | 172 | 4E+05 | S170 | MON/IMU//ALD/PVN | | CM85835-1Y-0H-05Y-4M-0Y-0SY-0SY | |
| 277 | 092 | 4E+06 |  | TAURUM | | CM92066-J-0BRA | |
| 278 | 270 | 6E+06 |  | CHONTE | | CGSS03B00169S-099M-099Y-099M-17WGY-0B | |
| 279 | 272 | 6E+06 | S170 | WHEAR/KIRITATI/3/C80.1/3*BATAVIA//2*WBLL1 | | CGSS03B00077T-099Y-099M-099Y-099M-47WGY-0B | |
| 280 | 223 | 4E+06 |  | SERI*3//RL6010/4*YR/3/PASTOR/4/BAV92 | | CMSS96M05696T-040Y-20M-010SY-010M-010SY-6M-0Y | |
| 281 | 081 | 4E+06 |  | W462//VEE/KOEL/3/PEG//MRL/BUC | | CMBW91M03389T-0TOPY-13M-1Y-010M-4KBY-3KBY-0M-0KBY-2PR-0B-1PR-010B-0Y-0HTY | |
| 282 | 242 | 5E+06 | S170 | PASTOR//MILAN/KAUZ/3/VEE/PJN//2*TUI | | CMSS97M03120T-040Y-040M-040SY-030M-040SY-18M-0Y-0SY | |
| 283 | 030 | 4E+05 | S170 | RHEA | | TE82.0009-18Y-025H-0Y-3M-0Y-0HTY | |
| 284 | 208 | 4E+06 |  | ATTILA*2/PBW65 | | CGSS96B00123F-099M-037Y-099M-26Y-0B-0SY | |
| 285 | 166 | 3E+05 | S170 | ESDA/4/BD120/3/GTA/MXP//RUFF/FGO | | CMBW89M5209-2M-010Y-010M-1Y-0M-0SY | |
| 286 | 294 | 5E+06 |  | BETTY/3/CHEN/AE.SQ//2*OPATA | | CMSW00WM00150S-040M-040Y-030M-030ZTM-8ZTY-0M-0SY | |
| 287 | 249 | 5E+06 | S170 | RL6043/4*NAC//2*PASTOR | | CMSS98M00790M-040Y-0100M-040Y-020M-040SY-2M-0Y-0SY | |
| 288 | 143 | 66363 | S170 | GRANERO INTA | | CM49641-9Y-1M-2Y-3Y-0M-1P-0P-0ARG | |
| 289 | 022 | 6E+05 | S170 | AZ//KAL/BB/3/PGO | | CM98924-0M-11Y-030M-030M-1Y-0Y-0HTY | |
| 290 | 079 | 4E+06 |  | SHA3/SERI//SHA4/LIRA/3/CHIR1/4/SHA7//PRL/VEE#6/3/FASAN | | CMSS96M05726T-040Y-15M-010Y-010M-020PR-2B-0Y-0HTY | |
| 291 | 075 | 4E+06 | S170 | KAUZ//BOW/NKT | | CMSS92Y02933S-20Y-015M-010Y-010Y-2M-0Y-0HTY | |
| 292 | 157 | 9E+05 | S170 | DUCULA//HUI/TUB/3/CAZO | | CMBW89M7554-0TOPY-030M-25Y-010M-0Y-0SY | |
| 293 | 190 | 2E+06 |  | MNCH/3*BCN | | CMBW90Y5756-0TOPM-14Y-010M-010M-010Y-5M-015Y-0Y-0HTY-0SY | |
| 294 | 069 | 1E+06 |  | RABE/2*MO88 | | CMSS92Y01634T-18Y-010M-010Y-010Y-2M-0Y-0HTY | |
| 295 | 125 | 41830 | S170 | BB//TOB/CNO67/3/HUAC/4/TI-R/3/BB/PL//SX | | CM62287-5M-1Y-1M-1Y-1M-0Y | |
| 296 | 085 | 4E+06 | S170 | CMH84.3379/CMH78.578//MILAN | | CMSS93Y00628S-7Y-010Y-010M-010Y-10M-0Y-3KBY-0KBY-0M-0HTY | |
| 297 | 181 | 2E+06 |  | NS-732/HER//KAUZ | | ICW91.0253-0TS-1AP-0TS-0AP-0SY | |
| 298 | 262 | 5E+06 | S170 | OASIS/5*BORL95/5/CNDO/R143//ENTE/MEXI75/3/AE.SQ/4/2*OCI | | CMSS98Y04800S-020Y-030M-020Y-040M-31Y-1M-0Y | |
| 299 | 101 | 5E+06 |  | ATTILA/3*BCN//BAV92/3/TILHI | | CMSS97M04169T-040Y-040M-020Y-030M-015Y-17M-2Y-3M-0Y | |
| Control 1- code= 299 | | | Name= Pishtaz Origin; Iran | | Alvand//Aldan/Ias | | |
| Control 2- code = 300 | | | Name= ghods Origin; Iran | | Rsh/ /Wt/</Nor >/K <*Q//Fn/ /Ptr/Y/Omid//Kal/Bb | | |
| Control 3- code = 301 | | | Name= roshan Origin; Iran | | A selection of landraces of Isfahan, Iran. | |  |

| **S2 Table.** Values of stress tolerance and sensitivity indices for all studied genotypes. The genotypes included in this study comprise a sub-population of the Wheat Association Mapping Initiative (WAMI), consisting of 153 advanced elite lines along with three Iranian cultivars used as controls (Pishtaz = 299, Qhods = 300, and Roshan = 301). The Gen column lists the genotypes, with their pedigree information provided in Supplementary Table 1 of this supplementary material. | | | | | | | | | | | | |
| --- | --- | --- | --- | --- | --- | --- | --- | --- | --- | --- | --- | --- |
| Gen | YP | YS | Tol | GMP | MP | YSI | HSI | STI | YI | HM | MRP | PYR |
| 1 | 908.75 | 732.50 | 176.25 | 13.28 | 820.6 | 0.81 | 0.77 | 0.82 | 1.09 | 811.16 | 2.10 | 19.39 |
| 3 | 1079.38 | 681.00 | 398.38 | 19.96 | 880.2 | 0.63 | 1.47 | 0.91 | 1.01 | 835.11 | 2.21 | 36.91 |
| 4 | 961.00 | 683.94 | 277.06 | 16.65 | 822.5 | 0.71 | 1.15 | 0.81 | 1.01 | 799.14 | 2.08 | 28.83 |
| 5 | 1046.25 | 834.31 | 211.94 | 14.56 | 940.3 | 0.80 | 0.81 | 1.08 | 1.24 | 928.33 | 2.40 | 20.26 |
| 8 | 1050.00 | 654.80 | 395.20 | 19.88 | 852.4 | 0.62 | 1.50 | 0.85 | 0.97 | 806.60 | 2.14 | 37.64 |
| 9 | 981.25 | 760.88 | 220.37 | 14.84 | 871.1 | 0.78 | 0.90 | 0.92 | 1.13 | 857.13 | 2.22 | 22.46 |
| 11 | 882.00 | 654.96 | 227.04 | 15.07 | 768.5 | 0.74 | 1.03 | 0.71 | 0.97 | 751.71 | 1.95 | 25.74 |
| 12 | 923.88 | 653.72 | 270.15 | 16.44 | 788.8 | 0.71 | 1.17 | 0.75 | 0.97 | 765.67 | 2.00 | 29.24 |
| 17 | 803.50 | 459.77 | 343.73 | 18.54 | 631.6 | 0.57 | 1.71 | 0.46 | 0.68 | 584.87 | 1.57 | 42.78 |
| 19 | 736.25 | 529.25 | 207.00 | 14.39 | 632.8 | 0.72 | 1.12 | 0.48 | 0.78 | 615.82 | 1.60 | 28.12 |
| 20 | 738.88 | 639.25 | 99.63 | 9.98 | 689.1 | 0.87 | 0.54 | 0.58 | 0.95 | 685.46 | 1.77 | 13.48 |
| 21 | 871.88 | 610.62 | 261.26 | 16.16 | 741.2 | 0.70 | 1.20 | 0.66 | 0.91 | 718.23 | 1.87 | 29.96 |
| 22 | 734.25 | 646.82 | 87.43 | 9.35 | 690.5 | 0.88 | 0.48 | 0.59 | 0.96 | 687.77 | 1.78 | 11.91 |
| 23 | 915.63 | 717.00 | 198.63 | 14.09 | 816.3 | 0.78 | 0.87 | 0.81 | 1.06 | 804.23 | 2.08 | 21.69 |
| 24 | 817.00 | 599.22 | 217.78 | 14.76 | 708.1 | 0.73 | 1.06 | 0.61 | 0.89 | 691.36 | 1.80 | 26.66 |
| 25 | 892.00 | 640.63 | 251.38 | 15.85 | 766.3 | 0.72 | 1.12 | 0.71 | 0.95 | 745.70 | 1.94 | 28.18 |
| 27 | 950.00 | 745.00 | 205.00 | 14.32 | 847.5 | 0.78 | 0.86 | 0.87 | 1.10 | 835.10 | 2.16 | 21.58 |
| 30 | 941.88 | 674.75 | 267.13 | 16.34 | 808.3 | 0.72 | 1.13 | 0.79 | 1.00 | 786.24 | 2.05 | 28.36 |
| 32 | 823.13 | 580.00 | 243.13 | 15.59 | 701.6 | 0.70 | 1.18 | 0.59 | 0.86 | 680.50 | 1.78 | 29.54 |
| 33 | 769.38 | 551.54 | 217.84 | 14.76 | 660.5 | 0.72 | 1.13 | 0.52 | 0.82 | 642.49 | 1.67 | 28.31 |
| 34 | 752.75 | 526.41 | 226.34 | 15.04 | 639.6 | 0.70 | 1.20 | 0.49 | 0.78 | 619.56 | 1.62 | 30.07 |
| 36 | 821.50 | 519.58 | 301.92 | 17.38 | 670.5 | 0.63 | 1.47 | 0.53 | 0.77 | 636.56 | 1.68 | 36.75 |
| 40 | 715.00 | 622.50 | 92.50 | 9.62 | 668.8 | 0.87 | 0.52 | 0.55 | 0.92 | 665.55 | 1.72 | 12.94 |
| 41 | 786.50 | 619.75 | 166.75 | 12.91 | 703.1 | 0.79 | 0.85 | 0.60 | 0.92 | 693.24 | 1.79 | 21.20 |
| 44 | 718.25 | 666.25 | 52.00 | 7.21 | 692.3 | 0.93 | 0.29 | 0.59 | 0.99 | 691.27 | 1.79 | 7.24 |
| 45 | 765.63 | 690.25 | 75.38 | 8.68 | 727.9 | 0.90 | 0.39 | 0.65 | 1.02 | 725.99 | 1.87 | 9.84 |
| 50 | 808.59 | 721.25 | 87.34 | 9.35 | 764.9 | 0.89 | 0.43 | 0.72 | 1.07 | 762.43 | 1.97 | 10.80 |
| 51 | 827.50 | 648.89 | 178.61 | 13.36 | 738.2 | 0.78 | 0.86 | 0.66 | 0.96 | 727.39 | 1.88 | 21.58 |
| 52 | 959.84 | 746.25 | 213.59 | 14.61 | 853.0 | 0.78 | 0.89 | 0.89 | 1.11 | 839.68 | 2.17 | 22.25 |
| 53 | 758.50 | 575.00 | 183.50 | 13.55 | 666.8 | 0.76 | 0.97 | 0.54 | 0.85 | 654.12 | 1.70 | 24.19 |
| 54 | 827.50 | 697.44 | 130.06 | 11.40 | 762.5 | 0.84 | 0.63 | 0.71 | 1.03 | 756.92 | 1.95 | 15.72 |
| 55 | 780.63 | 682.43 | 98.20 | 9.91 | 731.5 | 0.87 | 0.50 | 0.66 | 1.01 | 728.23 | 1.88 | 12.58 |
| 57 | 826.38 | 723.50 | 102.88 | 10.14 | 774.9 | 0.88 | 0.50 | 0.74 | 1.07 | 771.52 | 1.99 | 12.45 |
| 58 | 865.75 | 732.50 | 133.25 | 11.54 | 799.1 | 0.85 | 0.61 | 0.78 | 1.09 | 793.57 | 2.05 | 15.39 |
| 61 | 863.13 | 711.25 | 151.88 | 12.32 | 787.2 | 0.82 | 0.70 | 0.76 | 1.05 | 779.86 | 2.01 | 17.60 |
| 64 | 814.38 | 674.36 | 140.01 | 11.83 | 744.4 | 0.83 | 0.69 | 0.68 | 1.00 | 737.78 | 1.91 | 17.19 |
| 65 | 821.88 | 692.50 | 129.38 | 11.37 | 757.2 | 0.84 | 0.63 | 0.70 | 1.03 | 751.66 | 1.94 | 15.74 |
| 66 | 817.50 | 649.25 | 168.25 | 12.97 | 733.4 | 0.79 | 0.82 | 0.66 | 0.96 | 723.73 | 1.87 | 20.58 |
| 69 | 800.00 | 725.00 | 75.00 | 8.66 | 762.5 | 0.91 | 0.37 | 0.72 | 1.07 | 760.66 | 1.96 | 9.38 |
| 71 | 923.00 | 775.00 | 148.00 | 12.17 | 849.0 | 0.84 | 0.64 | 0.88 | 1.15 | 842.55 | 2.18 | 16.03 |
| 72 | 910.00 | 593.25 | 316.75 | 17.80 | 751.6 | 0.65 | 1.39 | 0.67 | 0.88 | 718.25 | 1.89 | 34.81 |
| 73 | 906.25 | 706.50 | 199.75 | 14.13 | 806.4 | 0.78 | 0.88 | 0.79 | 1.05 | 794.00 | 2.05 | 22.04 |
| 74 | 988.00 | 862.50 | 125.50 | 11.20 | 925.3 | 0.87 | 0.51 | 1.05 | 1.28 | 920.99 | 2.38 | 12.70 |
| 75 | 979.75 | 898.75 | 81.00 | 9.00 | 939.3 | 0.92 | 0.33 | 1.09 | 1.33 | 937.50 | 2.42 | 8.27 |
| 76 | 791.79 | 638.39 | 153.40 | 12.39 | 715.1 | 0.81 | 0.77 | 0.62 | 0.95 | 706.86 | 1.83 | 19.37 |
| 79 | 823.25 | 658.59 | 164.66 | 12.83 | 740.9 | 0.80 | 0.80 | 0.67 | 0.98 | 731.77 | 1.89 | 20.00 |
| 80 | 821.88 | 724.97 | 96.91 | 9.84 | 773.4 | 0.88 | 0.47 | 0.74 | 1.07 | 770.39 | 1.99 | 11.79 |
| 81 | 1006.88 | 688.50 | 318.38 | 17.84 | 847.7 | 0.68 | 1.26 | 0.86 | 1.02 | 817.79 | 2.14 | 31.62 |
| 82 | 1099.25 | 753.75 | 345.50 | 18.59 | 926.5 | 0.69 | 1.25 | 1.02 | 1.12 | 894.29 | 2.34 | 31.43 |
| 83 | 852.50 | 720.40 | 132.10 | 11.49 | 786.5 | 0.85 | 0.62 | 0.76 | 1.07 | 780.90 | 2.02 | 15.50 |
| 84 | 746.75 | 602.29 | 144.46 | 12.02 | 674.5 | 0.81 | 0.77 | 0.56 | 0.89 | 666.79 | 1.72 | 19.34 |
| 85 | 914.00 | 477.25 | 436.75 | 20.90 | 695.6 | 0.52 | 1.91 | 0.54 | 0.71 | 627.07 | 1.72 | 47.78 |
| 88 | 790.00 | 611.09 | 178.91 | 13.38 | 700.5 | 0.77 | 0.90 | 0.60 | 0.91 | 689.12 | 1.78 | 22.65 |
| 90 | 899.50 | 688.75 | 210.75 | 14.52 | 794.1 | 0.77 | 0.93 | 0.77 | 1.02 | 780.14 | 2.02 | 23.43 |
| 92 | 835.00 | 739.00 | 96.00 | 9.80 | 787.0 | 0.89 | 0.46 | 0.76 | 1.10 | 784.07 | 2.02 | 11.50 |
| 93 | 919.00 | 766.50 | 152.50 | 12.35 | 842.8 | 0.83 | 0.66 | 0.87 | 1.14 | 835.85 | 2.16 | 16.59 |
| 95 | 998.25 | 745.15 | 253.10 | 15.91 | 871.7 | 0.75 | 1.01 | 0.92 | 1.10 | 853.33 | 2.21 | 25.35 |
| 98 | 786.88 | 612.50 | 174.38 | 13.21 | 699.7 | 0.78 | 0.88 | 0.60 | 0.91 | 688.82 | 1.78 | 22.16 |
| 100 | 821.75 | 636.94 | 184.81 | 13.59 | 729.3 | 0.78 | 0.90 | 0.65 | 0.94 | 717.64 | 1.86 | 22.49 |
| 101 | 913.13 | 648.19 | 264.93 | 16.28 | 780.7 | 0.71 | 1.16 | 0.73 | 0.96 | 758.18 | 1.98 | 29.01 |
| 103 | 868.75 | 572.19 | 296.56 | 17.22 | 720.5 | 0.66 | 1.36 | 0.61 | 0.85 | 689.95 | 1.81 | 34.14 |
| 104 | 1021.88 | 668.88 | 353.00 | 18.79 | 845.4 | 0.65 | 1.38 | 0.84 | 0.99 | 808.52 | 2.13 | 34.54 |
| 106 | 812.38 | 747.34 | 65.04 | 8.06 | 779.9 | 0.92 | 0.32 | 0.75 | 1.11 | 778.50 | 2.01 | 8.01 |
| 108 | 889.00 | 695.75 | 193.25 | 13.90 | 792.4 | 0.78 | 0.87 | 0.76 | 1.03 | 780.59 | 2.02 | 21.74 |
| 114 | 826.25 | 707.50 | 118.75 | 10.90 | 766.9 | 0.86 | 0.57 | 0.72 | 1.05 | 762.28 | 1.97 | 14.37 |
| 116 | 742.75 | 548.75 | 194.00 | 13.93 | 645.8 | 0.74 | 1.04 | 0.50 | 0.81 | 631.18 | 1.64 | 26.12 |
| 118 | 969.75 | 727.00 | 242.75 | 15.58 | 848.4 | 0.75 | 1.00 | 0.87 | 1.08 | 831.01 | 2.16 | 25.03 |
| 121 | 1101.75 | 706.52 | 395.23 | 19.88 | 904.1 | 0.64 | 1.43 | 0.96 | 1.05 | 860.94 | 2.27 | 35.87 |
| 122 | 911.50 | 708.85 | 202.65 | 14.24 | 810.2 | 0.78 | 0.89 | 0.80 | 1.05 | 797.50 | 2.06 | 22.23 |
| 124 | 1068.50 | 646.45 | 422.05 | 20.54 | 857.5 | 0.61 | 1.58 | 0.85 | 0.96 | 805.54 | 2.15 | 39.50 |
| 125 | 1092.50 | 814.50 | 278.00 | 16.67 | 953.5 | 0.75 | 1.02 | 1.10 | 1.21 | 933.24 | 2.42 | 25.45 |
| 126 | 1094.49 | 754.18 | 340.30 | 18.45 | 924.3 | 0.69 | 1.24 | 1.02 | 1.12 | 893.01 | 2.33 | 31.09 |
| 127 | 856.25 | 648.75 | 207.50 | 14.40 | 752.5 | 0.76 | 0.97 | 0.69 | 0.96 | 738.20 | 1.91 | 24.23 |
| 130 | 828.88 | 708.75 | 120.13 | 10.96 | 768.8 | 0.86 | 0.58 | 0.73 | 1.05 | 764.12 | 1.97 | 14.49 |
| 131 | 948.25 | 722.75 | 225.50 | 15.02 | 835.5 | 0.76 | 0.95 | 0.85 | 1.07 | 820.28 | 2.13 | 23.78 |
| 139 | 898.00 | 800.00 | 98.00 | 9.90 | 849.0 | 0.89 | 0.44 | 0.89 | 1.19 | 846.17 | 2.18 | 10.91 |
| 140 | 931.00 | 771.00 | 160.00 | 12.65 | 851.0 | 0.83 | 0.69 | 0.89 | 1.14 | 843.48 | 2.18 | 17.19 |
| 143 | 959.25 | 772.75 | 186.50 | 13.66 | 866.0 | 0.81 | 0.78 | 0.92 | 1.15 | 855.96 | 2.21 | 19.44 |
| 145 | 1002.50 | 681.25 | 321.25 | 17.92 | 841.9 | 0.68 | 1.28 | 0.84 | 1.01 | 811.23 | 2.12 | 32.04 |
| 147 | 1008.75 | 680.42 | 328.33 | 18.12 | 844.6 | 0.67 | 1.30 | 0.85 | 1.01 | 812.67 | 2.13 | 32.55 |
| 148 | 1015.38 | 723.75 | 291.63 | 17.08 | 869.6 | 0.71 | 1.15 | 0.91 | 1.07 | 845.11 | 2.20 | 28.72 |
| 151 | 781.68 | 633.45 | 148.23 | 12.18 | 707.6 | 0.81 | 0.76 | 0.61 | 0.94 | 699.80 | 1.81 | 18.96 |
| 152 | 794.63 | 696.75 | 97.88 | 9.89 | 745.7 | 0.88 | 0.49 | 0.68 | 1.03 | 742.48 | 1.92 | 12.32 |
| 156 | 979.50 | 676.88 | 302.62 | 17.40 | 828.2 | 0.69 | 1.23 | 0.82 | 1.00 | 800.54 | 2.09 | 30.90 |
| 160 | 1098.63 | 807.50 | 291.13 | 17.06 | 953.1 | 0.74 | 1.06 | 1.10 | 1.20 | 930.83 | 2.42 | 26.50 |
| 161 | 963.25 | 765.24 | 198.01 | 14.07 | 864.2 | 0.79 | 0.82 | 0.91 | 1.13 | 852.90 | 2.21 | 20.56 |
| 162 | 922.25 | 748.68 | 173.57 | 13.17 | 835.5 | 0.81 | 0.75 | 0.85 | 1.11 | 826.45 | 2.14 | 18.82 |
| 164 | 1186.13 | 840.86 | 345.26 | 18.58 | 1013.5 | 0.71 | 1.16 | 1.23 | 1.25 | 984.09 | 2.57 | 29.11 |
| 166 | 940.00 | 739.00 | 201.00 | 14.18 | 839.5 | 0.79 | 0.85 | 0.86 | 1.10 | 827.47 | 2.14 | 21.38 |
| 167 | 1141.13 | 806.25 | 334.88 | 18.30 | 973.7 | 0.71 | 1.17 | 1.14 | 1.20 | 944.89 | 2.46 | 29.35 |
| 169 | 989.25 | 764.54 | 224.71 | 14.99 | 876.9 | 0.77 | 0.91 | 0.93 | 1.13 | 862.50 | 2.23 | 22.72 |
| 170 | 1069.25 | 701.25 | 368.00 | 19.18 | 885.3 | 0.66 | 1.37 | 0.93 | 1.04 | 847.01 | 2.23 | 34.42 |
| 171 | 925.00 | 701.41 | 223.59 | 14.95 | 813.2 | 0.76 | 0.96 | 0.80 | 1.04 | 797.84 | 2.07 | 24.17 |
| 172 | 1125.00 | 711.00 | 414.00 | 20.35 | 918.0 | 0.63 | 1.47 | 0.99 | 1.05 | 871.32 | 2.30 | 36.80 |
| 173 | 933.63 | 702.75 | 230.88 | 15.19 | 818.2 | 0.75 | 0.99 | 0.81 | 1.04 | 801.90 | 2.08 | 24.73 |
| 174 | 837.75 | 730.25 | 107.50 | 10.37 | 784.0 | 0.87 | 0.51 | 0.76 | 1.08 | 780.31 | 2.01 | 12.83 |
| 176 | 879.38 | 673.57 | 205.80 | 14.35 | 776.5 | 0.77 | 0.93 | 0.73 | 1.00 | 762.84 | 1.98 | 23.40 |
| 177 | 999.38 | 731.50 | 267.88 | 16.37 | 865.4 | 0.73 | 1.07 | 0.90 | 1.08 | 844.71 | 2.20 | 26.80 |
| 179 | 883.13 | 529.31 | 353.81 | 18.81 | 706.2 | 0.60 | 1.60 | 0.58 | 0.78 | 661.90 | 1.77 | 40.06 |
| 182 | 1006.25 | 643.21 | 363.04 | 19.05 | 824.7 | 0.64 | 1.44 | 0.80 | 0.95 | 784.78 | 2.07 | 36.08 |
| 184 | 821.75 | 674.58 | 147.17 | 12.13 | 748.2 | 0.82 | 0.71 | 0.69 | 1.00 | 740.93 | 1.91 | 17.91 |
| 185 | 1007.50 | 833.36 | 174.14 | 13.20 | 920.4 | 0.83 | 0.69 | 1.04 | 1.24 | 912.19 | 2.36 | 17.28 |
| 186 | 974.25 | 776.31 | 197.94 | 14.07 | 875.3 | 0.80 | 0.81 | 0.93 | 1.15 | 864.09 | 2.23 | 20.32 |
| 187 | 804.75 | 686.75 | 118.00 | 10.86 | 745.8 | 0.85 | 0.59 | 0.68 | 1.02 | 741.08 | 1.91 | 14.66 |
| 194 | 1001.25 | 654.12 | 347.13 | 18.63 | 827.7 | 0.65 | 1.38 | 0.81 | 0.97 | 791.29 | 2.08 | 34.67 |
| 195 | 1000.00 | 522.50 | 477.50 | 21.85 | 761.3 | 0.52 | 1.91 | 0.65 | 0.77 | 686.37 | 1.89 | 47.75 |
| 201 | 1052.00 | 727.75 | 324.25 | 18.01 | 889.9 | 0.69 | 1.23 | 0.95 | 1.08 | 860.34 | 2.25 | 30.82 |
| 203 | 755.81 | 600.75 | 155.06 | 12.45 | 678.3 | 0.79 | 0.82 | 0.56 | 0.89 | 669.42 | 1.73 | 20.52 |
| 205 | 1021.75 | 685.00 | 336.75 | 18.35 | 853.4 | 0.67 | 1.31 | 0.87 | 1.02 | 820.15 | 2.15 | 32.96 |
| 208 | 1045.00 | 753.99 | 291.01 | 17.06 | 899.5 | 0.72 | 1.11 | 0.97 | 1.12 | 875.96 | 2.28 | 27.85 |
| 209 | 945.63 | 667.25 | 278.38 | 16.68 | 806.4 | 0.71 | 1.17 | 0.78 | 0.99 | 782.41 | 2.04 | 29.44 |
| 211 | 981.13 | 695.63 | 285.50 | 16.90 | 838.4 | 0.71 | 1.16 | 0.84 | 1.03 | 814.07 | 2.12 | 29.10 |
| 212 | 853.75 | 695.92 | 157.83 | 12.56 | 774.8 | 0.82 | 0.74 | 0.73 | 1.03 | 766.80 | 1.98 | 18.49 |
| 214 | 742.50 | 608.75 | 133.75 | 11.57 | 675.6 | 0.82 | 0.72 | 0.56 | 0.90 | 669.01 | 1.73 | 18.01 |
| 215 | 954.88 | 685.00 | 269.88 | 16.43 | 819.9 | 0.72 | 1.13 | 0.81 | 1.02 | 797.73 | 2.08 | 28.26 |
| 220 | 933.13 | 717.50 | 215.63 | 14.68 | 825.3 | 0.77 | 0.92 | 0.83 | 1.06 | 811.23 | 2.10 | 23.11 |
| 222 | 773.25 | 627.25 | 146.00 | 12.08 | 700.3 | 0.81 | 0.75 | 0.60 | 0.93 | 692.64 | 1.79 | 18.88 |
| 225 | 743.00 | 575.69 | 167.31 | 12.93 | 659.3 | 0.77 | 0.90 | 0.53 | 0.85 | 648.73 | 1.68 | 22.52 |
| 226 | 1013.75 | 632.15 | 381.60 | 19.53 | 823.0 | 0.62 | 1.50 | 0.79 | 0.94 | 778.72 | 2.06 | 37.64 |
| 228 | 964.50 | 725.00 | 239.50 | 15.48 | 844.8 | 0.75 | 0.99 | 0.86 | 1.07 | 827.77 | 2.15 | 24.83 |
| 229 | 915.63 | 684.50 | 231.13 | 15.20 | 800.1 | 0.75 | 1.01 | 0.77 | 1.01 | 783.37 | 2.03 | 25.24 |
| 230 | 1013.00 | 681.89 | 331.11 | 18.20 | 847.4 | 0.67 | 1.30 | 0.85 | 1.01 | 815.10 | 2.14 | 32.69 |
| 232 | 941.38 | 723.50 | 217.88 | 14.76 | 832.4 | 0.77 | 0.92 | 0.84 | 1.07 | 818.18 | 2.12 | 23.14 |
| 233 | 908.75 | 728.86 | 179.89 | 13.41 | 818.8 | 0.80 | 0.79 | 0.82 | 1.08 | 808.92 | 2.09 | 19.80 |
| 234 | 961.25 | 703.25 | 258.00 | 16.06 | 832.3 | 0.73 | 1.07 | 0.84 | 1.04 | 812.25 | 2.11 | 26.84 |
| 235 | 891.25 | 757.50 | 133.75 | 11.57 | 824.4 | 0.85 | 0.60 | 0.83 | 1.12 | 818.95 | 2.11 | 15.01 |
| 240 | 915.00 | 738.11 | 176.89 | 13.30 | 826.6 | 0.81 | 0.77 | 0.83 | 1.09 | 817.09 | 2.11 | 19.33 |
| 241 | 810.25 | 668.85 | 141.40 | 11.89 | 739.6 | 0.83 | 0.70 | 0.67 | 0.99 | 732.79 | 1.89 | 17.45 |
| 242 | 872.50 | 737.00 | 135.50 | 11.64 | 804.8 | 0.84 | 0.62 | 0.79 | 1.09 | 799.05 | 2.06 | 15.53 |
| 247 | 854.25 | 659.92 | 194.33 | 13.94 | 757.1 | 0.77 | 0.91 | 0.70 | 0.98 | 744.62 | 1.93 | 22.75 |
| 248 | 796.25 | 670.00 | 126.25 | 11.24 | 733.1 | 0.84 | 0.63 | 0.66 | 0.99 | 727.69 | 1.88 | 15.86 |
| 249 | 875.50 | 624.25 | 251.25 | 15.85 | 749.9 | 0.71 | 1.14 | 0.68 | 0.93 | 728.83 | 1.90 | 28.70 |
| 255 | 995.63 | 686.25 | 309.38 | 17.59 | 840.9 | 0.69 | 1.24 | 0.84 | 1.02 | 812.48 | 2.12 | 31.07 |
| 260 | 998.00 | 774.00 | 224.00 | 14.97 | 886.0 | 0.78 | 0.90 | 0.95 | 1.15 | 871.84 | 2.26 | 22.44 |
| 263 | 1124.75 | 670.50 | 454.25 | 21.31 | 897.6 | 0.60 | 1.61 | 0.93 | 0.99 | 840.16 | 2.24 | 40.39 |
| 265 | 959.88 | 601.88 | 358.00 | 18.92 | 780.9 | 0.63 | 1.49 | 0.71 | 0.89 | 739.84 | 1.96 | 37.30 |
| 266 | 1011.25 | 650.58 | 360.67 | 18.99 | 830.9 | 0.64 | 1.42 | 0.81 | 0.96 | 791.78 | 2.09 | 35.67 |
| 271 | 1040.50 | 685.75 | 354.75 | 18.83 | 863.1 | 0.66 | 1.36 | 0.88 | 1.02 | 826.67 | 2.17 | 34.09 |
| 272 | 885.00 | 753.28 | 131.72 | 11.48 | 819.1 | 0.85 | 0.59 | 0.82 | 1.12 | 813.84 | 2.10 | 14.88 |
| 273 | 860.75 | 745.25 | 115.50 | 10.75 | 803.0 | 0.87 | 0.54 | 0.79 | 1.10 | 798.85 | 2.06 | 13.42 |
| 274 | 951.25 | 740.00 | 211.25 | 14.53 | 845.6 | 0.78 | 0.89 | 0.87 | 1.10 | 832.43 | 2.15 | 22.21 |
| 276 | 733.25 | 551.58 | 181.67 | 13.48 | 642.4 | 0.75 | 0.99 | 0.50 | 0.82 | 629.57 | 1.63 | 24.78 |
| 279 | 963.75 | 620.36 | 343.39 | 18.53 | 792.1 | 0.64 | 1.42 | 0.74 | 0.92 | 754.84 | 1.99 | 35.63 |
| 283 | 749.88 | 645.00 | 104.88 | 10.24 | 697.4 | 0.86 | 0.56 | 0.60 | 0.96 | 693.49 | 1.79 | 13.99 |
| 285 | 861.25 | 640.50 | 220.75 | 14.86 | 750.9 | 0.74 | 1.02 | 0.68 | 0.95 | 734.65 | 1.91 | 25.63 |
| 287 | 813.00 | 618.82 | 194.18 | 13.93 | 715.9 | 0.76 | 0.95 | 0.62 | 0.92 | 702.74 | 1.82 | 23.88 |
| 288 | 647.38 | 572.50 | 74.88 | 8.65 | 609.9 | 0.88 | 0.46 | 0.46 | 0.85 | 607.64 | 1.57 | 11.57 |
| 289 | 721.99 | 566.87 | 155.12 | 12.45 | 644.4 | 0.79 | 0.86 | 0.51 | 0.84 | 635.09 | 1.64 | 21.48 |
| 291 | 787.00 | 587.75 | 199.25 | 14.12 | 687.4 | 0.75 | 1.01 | 0.57 | 0.87 | 672.94 | 1.75 | 25.32 |
| 292 | 804.38 | 494.50 | 309.88 | 17.60 | 649.4 | 0.61 | 1.54 | 0.49 | 0.73 | 612.47 | 1.63 | 38.52 |
| 295 | 746.25 | 479.03 | 267.22 | 16.35 | 612.6 | 0.64 | 1.43 | 0.44 | 0.71 | 583.50 | 1.54 | 35.81 |
| 296 | 854.50 | 440.25 | 414.25 | 20.35 | 647.4 | 0.52 | 1.93 | 0.46 | 0.65 | 581.11 | 1.60 | 48.48 |
| 298 | 1020.75 | 701.75 | 319.00 | 17.86 | 861.3 | 0.69 | 1.25 | 0.89 | 1.04 | 831.71 | 2.18 | 31.25 |
| 299 | 690.63 | 417.50 | 273.13 | 16.53 | 554.1 | 0.60 | 1.58 | 0.36 | 0.62 | 520.40 | 1.39 | 39.55 |
| 300 | 860.00 | 441.75 | 418.25 | 20.45 | 650.9 | 0.51 | 1.94 | 0.47 | 0.65 | 583.68 | 1.61 | 48.63 |
| 301 | 906.25 | 580.75 | 325.50 | 18.04 | 743.5 | 0.64 | 1.43 | 0.65 | 0.86 | 707.87 | 1.87 | 35.92 |
| Grain yield of genotypes under normal condition (Yp), Grain yield of genotypes under heat stress condition (Ys), Tolerance index (TOL), Yield stability index (YSI), Heat susceptibility index (HIS), Mean productivity (MP), Geometric mean productivity (GMP), Stress tolerance index (STI), Harmonic Mean (HM), Yield Index (YI), Mean relative performance (MRP), Percent yield Reduction (PYR). | | | | | | | | | | | | |

| **S3 Table.** Clustering of genotypes from the WAMI sub-population and control cultivars (additional genotype information is provided in Supplementary Table 1) into three groups: tolerant, semi-tolerant, and susceptible. Genotypes with HSI values < 0.8 were classified as tolerant, those with values between 0.8 and 1.2 as semi-tolerant, and those with values > 1.2 as susceptible genotypes. In several studies, other researchers have also utilized the HSI index to identify tolerant genotypes (Kumar et al. 2021; Sharma et al. 2023), and the results of our study align with their findings. | | | | | | | | |
| --- | --- | --- | --- | --- | --- | --- | --- | --- |
| **Susceptible Groups** | | **Semi-tolerant Groups** | | | | | **Tolerant Groups** | |
| **HSI** | **Genotype** | **HSI** | **Genotype** | **HSI** | **Genotype** | **HSI** | | **Genotype** |
| 1.47 | 3 | 1.16 | 211 | 1.15 | 4 | 0.77 | | 1 |
| 1.50 | 8 | 1.13 | 215 | 0.81 | 5 | 0.54 | | 20 |
| 1.71 | 17 | 0.92 | 220 | 0.90 | 9 | 0.48 | | 22 |
| 1.20 | 21 | 0.90 | 225 | 1.03 | 11 | 0.52 | | 40 |
| 1.47 | 36 | 0.99 | 228 | 1.17 | 12 | 0.29 | | 44 |
| 1.39 | 72 | 1.01 | 229 | 1.12 | 19 | 0.39 | | 45 |
| 1.26 | 81 | 0.92 | 232 | 0.87 | 23 | 0.43 | | 50 |
| 1.25 | 82 | 1.07 | 234 | 1.06 | 24 | 0.63 | | 54 |
| 1.91 | 85 | 0.91 | 247 | 1.12 | 25 | 0.50 | | 55 |
| 1.36 | 103 | 1.14 | 249 | 0.86 | 27 | 0.50 | | 57 |
| 1.38 | 104 | 0.90 | 260 | 1.13 | 30 | 0.61 | | 58 |
| 1.43 | 121 | 0.89 | 274 | 1.18 | 32 | 0.70 | | 61 |
| 1.58 | 124 | 0.99 | 276 | 1.13 | 33 | 0.69 | | 64 |
| 1.28 | 145 | 1.02 | 285 | 1.20 | 34 | 0.63 | | 65 |
| 1.30 | 147 | 0.95 | 287 | 0.85 | 41 | 0.37 | | 69 |
| 1.23 | 156 | 0.86 | 289 | 0.86 | 51 | 0.64 | | 71 |
| 1.37 | 170 | 1.01 | 291 | 0.89 | 52 | 0.51 | | 74 |
| 1.47 | 172 |  |  | 0.97 | 53 | 0.33 | | 75 |
| 1.60 | 179 |  |  | 0.82 | 66 | 0.77 | | 76 |
| 1.44 | 182 |  |  | 0.88 | 73 | 0.47 | | 80 |
| 1.38 | 194 |  |  | 0.80 | 79 | 0.62 | | 83 |
| 1.91 | 195 |  |  | 0.90 | 88 | 0.77 | | 84 |
| 1.23 | 201 |  |  | 0.93 | 90 | 0.46 | | 92 |
| 1.31 | 205 |  |  | 1.01 | 95 | 0.66 | | 93 |
| 1.50 | 226 |  |  | 0.88 | 98 | 0.32 | | 106 |
| 1.30 | 230 |  |  | 0.90 | 100 | 0.57 | | 114 |
| 1.24 | 255 |  |  | 1.16 | 101 | 0.58 | | 130 |
| 1.61 | 263 |  |  | 0.87 | 108 | 0.44 | | 139 |
| 1.49 | 265 |  |  | 1.04 | 116 | 0.69 | | 140 |
| 1.42 | 266 |  |  | 1.00 | 118 | 0.78 | | 143 |
| 1.36 | 271 |  |  | 0.89 | 122 | 0.76 | | 151 |
| 1.42 | 279 |  |  | 1.02 | 125 | 0.49 | | 152 |
| 1.54 | 292 |  |  | 1.24 | 126 | 0.75 | | 162 |
| 1.43 | 295 |  |  | 0.97 | 127 | 0.51 | | 174 |
| 1.93 | 296 |  |  | 0.95 | 131 | 0.71 | | 184 |
| 1.25 | 298 |  |  | 1.15 | 148 | 0.69 | | 185 |
| 1.58 | 299(C1) |  |  | 1.06 | 160 | 0.59 | | 187 |
| 1.94 | 300(C2) |  |  | 0.82 | 161 | 0.74 | | 212 |
| 1.43 | 301(C3) |  |  | 1.16 | 164 | 0.72 | | 214 |
|  |  |  |  | 0.85 | 166 | 0.75 | | 222 |
|  |  |  |  | 1.17 | 167 | 0.79 | | 233 |
|  |  |  |  | 0.91 | 169 | 0.60 | | 235 |
|  |  |  |  | 0.96 | 171 | 0.77 | | 240 |
|  |  |  |  | 0.99 | 173 | 0.70 | | 241 |
|  |  |  |  | 0.93 | 176 | 0.62 | | 242 |
|  |  |  |  | 1.07 | 177 | 0.63 | | 248 |
|  |  |  |  | 0.81 | 186 | 0.59 | | 272 |
|  |  |  |  | 0.82 | 203 | 0.54 | | 273 |
|  |  |  |  | 1.11 | 208 | 0.56 | | 283 |
|  |  |  |  | 1.17 | 209 | 0.46 | | 288 |

| **S4** **Table.** Analysis of variance (ANOVA) for agronomic and quality-related traits in the Wheat Association Mapping Initiative (WAMI) panel (153 genotypes and three control cultivars) evaluated under the fall sowing date (SD, normal environment). | | | | | | | | | |
| --- | --- | --- | --- | --- | --- | --- | --- | --- | --- |
| **Trait** |  |  | **Year (Y)** | **Rep (Y)** | **Genotype (G)** | **G* Y** | **Residuals** | **CV (%)** | **R²** |
|  | **DF** |  | 1 | 2 | 155 | 155 | 310 |  |  |
| **BY** |  | **Means of Squares** | 8788453^**^ | 105246 | 289719^**^ | 126469^**^ | 10314 | 3.71 | 0.95 |
| **DA** |  |  | 2060^**^ | 8.9647 | 9.81^**^ | 3.01^**^ | 0.70 | 0.52 | 0.94 |
| **DB** |  |  | 1157^**^ | 16.6314 | 13.92^**^ | 4.47^**^ | 0.85 | 0.88 | 0.93 |
| **DF** |  |  | 1025^**^ | 19.04 | 12.79^**^ | 4.12^**^ | 0.76 | 0.54 | 0.93 |
| **DM** |  |  | 2745^**^ | 3.04 | 10.82^**^ | 2.52^**^ | 1.18 | 0.56 | 0.92 |
| **GH** |  |  | 191^**^ | 1.61 | 1.59^**^ | 0.93^**^ | 0.48 | 1.58 | 0.79 |
| **GY** |  |  | 665339^*^ | 39723 | 47093^**^ | 15624^**^ | 2641 | 5.71 | 0.92 |
| **HI** |  |  | 68.2^ns^ | 11.34 | 44.8^**^ | 7.35^**^ | 1.87 | 4.10 | 0.93 |
| **PGP** |  |  | 19.93^ns^ | 3.06 | 0.39^**^ | 0.17^**^ | 0.09 | 2.36 | 0.79 |
| **PH** |  |  | 2823^**^ | 20.7 | 75.2^**^ | 27.7^**^ | 16.9 | 4.76 | 0.78 |
| **PL** |  |  | 33^ns^ | 42.4 | 14.2^**^ | 7.28^**^ | 3.61 | 6.80 | 0.76 |
| **SL** |  |  | 56.88^*^ | 1.75 | 1.95^**^ | 0.54^**^ | 0.27 | 5.98 | 0.84 |
| **TKW** |  |  | 64.20^ns^ | 7.15 | 57.4^**^ | 2.53^ns^ | 4.53 | 5.02 | 0.86 |
| **TW** |  |  | 0.23^ns^ | 0.50 | 13.9^**^ | 0.66^ns^ | 2.25 | 1.76 | 0.76 |
| **ZSV** |  |  | 1.91^ns^ | 20.88 | 0.98^*^ | 0.76^**^ | 0.23 | 1.49 | 0.81 |
| R² = coefficient of determination; CV (%) = coefficient of variation; * and ** indicate significance at p < 0.05 and p < 0.01, respectively. ns: non-significant. Days to booting (DB), Days to flowering (DF), Days to anthesis (DA), Days to maturity (DM), Plant height (PH), Peduncle length (PL), Thousand Kernel Weigh (TKW), Spike length (SL), Test weight (TW), Grain yield (GY), Biological yield (BY), Harvest index (HI), Percentage of grain protein (PGP), Zeleny index sedimentation volume (ZSV), Grain hardness (GH). | | | | | | | | | |

| **S5 Table.** Analysis of variance (ANOVA) for agronomic and quality-related traits in the Wheat Association Mapping Initiative (WAMI) panel (153 genotypes and three control cultivars) evaluated under the spring sowing date (SD, heat-stress environment). | | | | | | | | | | |
| --- | --- | --- | --- | --- | --- | --- | --- | --- | --- | --- |
| **Trait** |  |  | **Year (Y)** | **Rep (Y)** | **Genotype (G)** | **G* Y** | **Residual** | **CV (%)** | **R²** | |
|  | **DF** |  | 1 | 2 | 155 | 155 | 310 |  |  | |
| **BY** |  | **Means of Squares** | 8881025** | 55600ns | 259980** | 103962** | 22614 | 5.81 | 0.90 | |
| **DA** |  |  | 25.8ns | 8.08** | 6.14** | 0.93ns | 0.86 | 0.90 | 0.80 | |
| **DB** |  |  | 21303** | 22.7** | 16.8** | 10.5** | 2.09 | 1.77 | 0.97 | |
| **DF** |  |  | 4952** | 2.96ns | 11.3* | 8.19** | 1.06 | 1.18 | 0.96 | |
| **DM** |  |  | 5932** | 5.08* | 7.46 | 8.64** | 1.32 | 0.94 | 0.95 | |
| **GH** |  |  | 112ns | 48.8** | 11.3** | 2.05** | 0.95 | 2.27 | 0.88 | |
| **GY** |  |  | 810078** | 4973ns | 28587** | 7134** | 2077 | 6.75 | 0.90 | |
| **HI** |  |  | 15.83ns | 4.55ns | 15.8** | 3.08** | 1.88 | 5.26 | 0.83 | |
| **PGP** |  |  | 39.38ns | 5.45** | 0.61** | 0.21** | 0.08 | 2.29 | 0.86 | |
| **PH** |  |  | 88.05ns | 27.8* | 108** | 17.4** | 6.96 | 3.57 | 0.90 | |
| **PL** |  |  | 310* | 20.2** | 29.6** | 6.34** | 4.18 | 7.87 | 0.82 | |
| **SL** |  |  | 0.11ns | 0.80ns | 2.29** | 0.56** | 0.31 | 6.98 | 0.82 | |
| **TKW** |  |  | 137ns | 14.08** | 66.5** | 0.44ns | 2.01 | 3.91 | 0.94 | |
| **TW** |  |  | 181ns | 86.01** | 17.4** | 7.08** | 3.67 | 2.33 | 0.78 | |
| **ZSV** |  |  | 16.11ns | 5.55** | 1.30** | 0.43** | 0.30 | 1.72 | 0.75 | |
| R² = coefficient of determination; CV (%) = coefficient of variation; * and ** indicate significance at p < 0.05 and p < 0.01, respectively. ns: non-significant. Days to booting (DB), Days to flowering (DF), Days to anthesis (DA), Days to maturity (DM), Plant height (PH), Peduncle length (PL), Thousand Kernel Weigh (TKW), Spike length (SL), Test weight (TW), Grain yield (GY), Biological yield (BY), Harvest index (HI), Percentage of grain protein (PGP), Zeleny index sedimentation volume (ZSV), Grain hardness (GH). | | | | | | | | | |  |

| **S6 Table.** Mean values of grain yield (GY), thousand-kernel weight (TKW), and protein content (PGP) for the top 10 heat-tolerant genotypes in the Wheat Association Mapping Initiative (WAMI) panel (153 genotypes and three control cultivars) evaluated under normal (Fall SD) and heat-stress (Spring SD) environments, with LSD (0.05) mean separations. | | | | | | | | |
| --- | --- | --- | --- | --- | --- | --- | --- | --- |
| **Rank** | **Genotypes** | **WAMI ID** | **GY (Fall)**  **(g/m^2^)** | **GY (Spring) (g/m^2^)** | **TKW (Fall) (g)** | **TKW (Spring) (g)** | **PGP (Fall) %** | **PGP (Spring) %** |
| 1 | 75 | 104 | 979^ab^ | 898^a^ | 46.40^b^ | 36.64^c^ | 12.93^bcd^ | 12.58^c^ |
| 2 | 74 | 123 | 988^ab^ | 862^ab^ | 42.63^d^ | 36.52^c^ | 13.45^ab^ | 12.96^abc^ |
| 3 | 139 | 105 | 898^b^ | 800^cd^ | 45.19b^cd^ | 39.40^b^ | 12.69^cd^ | 11.99^d^ |
| 4 | 185 | 292 | 1007^ab^ | 833^bc^ | 37.14^e^ | 32.38^d^ | 12.52^d^ | 13.20^d^ |
| 5 | 5 | 039 | 1046^a^ | 834^bc^ | 52.04^a^ | 45.37^a^ | 12.78^cd^ | 12.63^c^ |
| 6 | 140 | 006 | 931^ab^ | 771^d^ | 38.72^e^ | 32.17^d^ | 13.15^abc^ | 12.87^bc^ |
| 7 | 71 | 271 | 923^ab^ | 775^cd^ | 47.35^b^ | 40.23^b^ | 13.08^bc^ | 13.45^a^ |
| 8 | 186 | 058 | 974^ab^ | 776^cd^ | 38.09^e^ | 32.27^d^ | 12.90^cd^ | 13.45^a^ |
| 9 | 143 | 204 | 959^ab^ | 772^cd^ | 43.24^cd^ | 37.37^c^ | 13.64^a^ | 13.20^ab^ |
| 10 | 93 | 035 | 919^b^ | 766^d^ | 46.24^bc^ | 39.6^b^ | 13.10^bc^ | 12.97^abc^ |
| LSD (0.05) | | | 123.8 | 62.13 | 3.14 | 1.81 | 0.053 | 0.50 |
| LSD (0.05) letters indicate significant differences within each column. GY = grain yield (g m⁻²), TKW = thousand-kernel weight (g), PGP = percentage of grain protein (%). | | | | | | | | |

**S7 Table.** Complete ranking of 156 wheat genotypes (153 WAMI lines plus three control cultivars) from the Wheat Association Mapping Initiative (WAMI) panel, evaluated under normal (NC; fall sowing) and heat-stress (HS; spring sowing) conditions, using a composite ranking approach based on three indices (HSI, MP, STI).

| **Rank** | **Eligible** | **HT_score** | **d_HSI** | **d_MP** | **d_STI** | **GMP** | **YSI** | **Gen** | **YP** | **YS** | **MP** | **HSI** | **STI** |
| --- | --- | --- | --- | --- | --- | --- | --- | --- | --- | --- | --- | --- | --- |
| 1 | 1 | 0.98 | 0.99 | 0.97 | 0.97 | 938.38 | 0.92 | 75 | 980 | 899 | 939 | 0.33 | 1.09 |
| 2 | 1 | 0.93 | 0.91 | 0.95 | 0.96 | 923.12 | 0.87 | 74 | 988 | 863 | 925 | 0.51 | 1.05 |
| 3 | 1 | 0.88 | 0.96 | 0.78 | 0.81 | 847.58 | 0.89 | 139 | 898 | 800 | 849 | 0.44 | 0.89 |
| 4 | 1 | 0.87 | 0.79 | 0.94 | 0.95 | 916.30 | 0.83 | 185 | 1008 | 833 | 920 | 0.69 | 1.04 |
| 5 | 1 | 0.82 | 0.67 | 0.97 | 0.97 | 934.29 | 0.80 | 5 | 1046 | 834 | 940 | 0.81 | 1.08 |
| 6 | 1 | 0.80 | 0.79 | 0.79 | 0.81 | 847.23 | 0.83 | 140 | 931 | 771 | 851 | 0.69 | 0.89 |
| 7 | 1 | 0.80 | 0.80 | 0.78 | 0.80 | 845.77 | 0.84 | 71 | 923 | 775 | 849 | 0.64 | 0.88 |
| 8 | 1 | 0.78 | 0.67 | 0.88 | 0.88 | 869.67 | 0.80 | 186 | 974 | 776 | 875 | 0.81 | 0.93 |
| 9 | 1 | 0.77 | 0.69 | 0.85 | 0.86 | 860.96 | 0.81 | 143 | 959 | 773 | 866 | 0.78 | 0.92 |
| 10 | 1 | 0.77 | 0.79 | 0.72 | 0.77 | 839.29 | 0.83 | 93 | 919 | 767 | 843 | 0.66 | 0.87 |
| 11 | 1 | 0.75 | 0.66 | 0.84 | 0.85 | 858.56 | 0.79 | 161 | 963 | 765 | 864 | 0.82 | 0.91 |
| 12 | 1 | 0.74 | 0.85 | 0.63 | 0.65 | 821.66 | 0.85 | 235 | 891 | 758 | 824 | 0.60 | 0.83 |
| 13 | 1 | 0.73 | 0.56 | 0.90 | 0.91 | 878.89 | 0.78 | 260 | 998 | 774 | 886 | 0.90 | 0.95 |
| 14 | 1 | 0.73 | 0.86 | 0.59 | 0.63 | 816.49 | 0.85 | 272 | 885 | 753 | 819 | 0.59 | 0.82 |
| 15 | 1 | 0.72 | 0.74 | 0.68 | 0.71 | 830.95 | 0.81 | 162 | 922 | 749 | 836 | 0.75 | 0.85 |
| 16 | 1 | 0.71 | 0.56 | 0.86 | 0.86 | 864.07 | 0.78 | 9 | 981 | 761 | 871 | 0.90 | 0.92 |
| 17 | 1 | 0.71 | 0.89 | 0.53 | 0.54 | 800.92 | 0.87 | 273 | 861 | 745 | 803 | 0.54 | 0.79 |
| 18 | 1 | 0.71 | 0.53 | 0.88 | 0.88 | 869.67 | 0.77 | 169 | 989 | 765 | 877 | 0.91 | 0.93 |
| 19 | 1 | 0.70 | 0.41 | 0.99 | 0.98 | 943.31 | 0.75 | 125 | 1093 | 815 | 954 | 1.02 | 1.10 |
| 20 | 1 | 0.70 | 0.58 | 0.81 | 0.81 | 846.33 | 0.78 | 52 | 960 | 746 | 853 | 0.89 | 0.89 |
| 21 | 1 | 0.70 | 0.63 | 0.76 | 0.77 | 841.28 | 0.78 | 27 | 950 | 745 | 848 | 0.86 | 0.87 |
| 22 | 1 | 0.68 | 0.83 | 0.54 | 0.54 | 801.89 | 0.84 | 242 | 873 | 737 | 805 | 0.62 | 0.79 |
| 23 | 1 | 0.68 | 0.39 | 0.98 | 0.98 | 941.88 | 0.74 | 160 | 1099 | 808 | 953 | 1.06 | 1.10 |
| 24 | 1 | 0.68 | 0.64 | 0.70 | 0.75 | 833.46 | 0.79 | 166 | 940 | 739 | 840 | 0.85 | 0.86 |
| 25 | 1 | 0.68 | 0.72 | 0.65 | 0.65 | 821.81 | 0.81 | 240 | 915 | 738 | 827 | 0.77 | 0.83 |
| 26 | 1 | 0.68 | 0.84 | 0.52 | 0.52 | 796.34 | 0.85 | 58 | 866 | 733 | 799 | 0.61 | 0.78 |
| 27 | 1 | 0.67 | 0.58 | 0.75 | 0.77 | 839.00 | 0.78 | 274 | 951 | 740 | 846 | 0.89 | 0.87 |
| 28 | 1 | 0.67 | 0.72 | 0.61 | 0.63 | 815.88 | 0.81 | 1 | 909 | 733 | 821 | 0.77 | 0.82 |
| 29 | 1 | 0.65 | 0.30 | 1.00 | 1.00 | 998.68 | 0.71 | 164 | 1186 | 841 | 1014 | 1.16 | 1.23 |
| 30 | 1 | 0.65 | 0.43 | 0.87 | 0.86 | 862.47 | 0.75 | 95 | 998 | 745 | 872 | 1.01 | 0.92 |
| 31 | 1 | 0.65 | 0.68 | 0.59 | 0.63 | 813.85 | 0.80 | 233 | 909 | 729 | 819 | 0.79 | 0.82 |
| 32 | 1 | 0.64 | 0.36 | 0.92 | 0.93 | 887.65 | 0.72 | 208 | 1045 | 754 | 900 | 1.11 | 0.97 |
| 33 | 1 | 0.64 | 0.28 | 0.99 | 0.99 | 959.19 | 0.71 | 167 | 1141 | 806 | 974 | 1.17 | 1.14 |
| 34 | 1 | 0.61 | 0.37 | 0.85 | 0.84 | 855.01 | 0.73 | 177 | 999 | 732 | 865 | 1.07 | 0.90 |
| 35 | 1 | 0.60 | 0.44 | 0.77 | 0.77 | 839.65 | 0.75 | 118 | 970 | 727 | 848 | 1.00 | 0.87 |
| 36 | 1 | 0.60 | 0.46 | 0.74 | 0.75 | 836.22 | 0.75 | 228 | 965 | 725 | 845 | 0.99 | 0.86 |
| 37 | 1 | 0.60 | 0.52 | 0.68 | 0.67 | 825.28 | 0.77 | 232 | 941 | 724 | 832 | 0.92 | 0.84 |
| 38 | 1 | 0.59 | 0.49 | 0.68 | 0.71 | 827.86 | 0.76 | 131 | 948 | 723 | 836 | 0.95 | 0.85 |
| 39 | 1 | 0.59 | 0.61 | 0.57 | 0.59 | 810.25 | 0.78 | 23 | 916 | 717 | 816 | 0.87 | 0.81 |
| 40 | 1 | 0.59 | 0.23 | 0.95 | 0.94 | 908.54 | 0.69 | 126 | 1094 | 754 | 924 | 1.24 | 1.02 |
| 41 | 1 | 0.59 | 0.22 | 0.96 | 0.94 | 910.25 | 0.69 | 82 | 1099 | 754 | 927 | 1.25 | 1.02 |
| 42 | 1 | 0.58 | 0.32 | 0.86 | 0.85 | 857.25 | 0.71 | 148 | 1015 | 724 | 870 | 1.15 | 0.91 |
| 43 | 1 | 0.58 | 0.52 | 0.64 | 0.65 | 818.24 | 0.77 | 220 | 933 | 718 | 825 | 0.92 | 0.83 |
| 44 | 1 | 0.58 | 0.25 | 0.91 | 0.91 | 874.98 | 0.69 | 201 | 1052 | 728 | 890 | 1.23 | 0.95 |
| 45 | 1 | 0.57 | 0.58 | 0.56 | 0.57 | 803.81 | 0.78 | 122 | 912 | 709 | 810 | 0.89 | 0.80 |
| 46 | 1 | 0.57 | 0.59 | 0.54 | 0.54 | 800.17 | 0.78 | 73 | 906 | 707 | 806 | 0.88 | 0.79 |
| 47 | 1 | 0.55 | 0.61 | 0.50 | 0.48 | 786.46 | 0.78 | 108 | 889 | 696 | 792 | 0.87 | 0.76 |
| 48 | 1 | 0.52 | 0.37 | 0.67 | 0.67 | 822.19 | 0.73 | 234 | 961 | 703 | 832 | 1.07 | 0.84 |
| 49 | 1 | 0.52 | 0.48 | 0.57 | 0.57 | 805.48 | 0.76 | 171 | 925 | 701 | 813 | 0.96 | 0.80 |
| 50 | 1 | 0.52 | 0.46 | 0.58 | 0.59 | 810.01 | 0.75 | 173 | 934 | 703 | 818 | 0.99 | 0.81 |
| 51 | 1 | 0.52 | 0.22 | 0.83 | 0.81 | 846.35 | 0.69 | 298 | 1021 | 702 | 861 | 1.25 | 0.89 |
| 52 | 1 | 0.51 | 0.50 | 0.51 | 0.51 | 787.10 | 0.77 | 90 | 900 | 689 | 794 | 0.93 | 0.77 |
| 53 | 1 | 0.49 | 0.30 | 0.70 | 0.67 | 826.14 | 0.71 | 211 | 981 | 696 | 838 | 1.16 | 0.84 |
| 54 | 1 | 0.48 | 0.21 | 0.77 | 0.75 | 832.61 | 0.68 | 81 | 1007 | 689 | 848 | 1.26 | 0.86 |
| 55 | 1 | 0.47 | 0.43 | 0.52 | 0.51 | 791.67 | 0.75 | 229 | 916 | 685 | 800 | 1.01 | 0.77 |
| 56 | 1 | 0.47 | 0.34 | 0.60 | 0.59 | 808.76 | 0.72 | 215 | 955 | 685 | 820 | 1.13 | 0.81 |
| 57 | 1 | 0.46 | 0.19 | 0.75 | 0.71 | 831.12 | 0.67 | 230 | 1013 | 682 | 847 | 1.30 | 0.85 |
| 58 | 1 | 0.46 | 0.23 | 0.71 | 0.67 | 826.59 | 0.69 | 255 | 996 | 686 | 841 | 1.24 | 0.84 |
| 59 | 1 | 0.46 | 0.32 | 0.61 | 0.59 | 810.72 | 0.71 | 4 | 961 | 684 | 823 | 1.15 | 0.81 |
| 60 | 1 | 0.46 | 0.19 | 0.73 | 0.71 | 828.48 | 0.67 | 147 | 1009 | 680 | 845 | 1.30 | 0.85 |
| 61 | 1 | 0.45 | 0.20 | 0.72 | 0.67 | 826.41 | 0.68 | 145 | 1003 | 681 | 842 | 1.28 | 0.84 |
| 62 | 1 | 0.44 | 0.25 | 0.66 | 0.63 | 814.25 | 0.69 | 156 | 980 | 677 | 828 | 1.23 | 0.82 |
| 63 | 1 | 0.44 | 0.34 | 0.55 | 0.54 | 797.20 | 0.72 | 30 | 942 | 675 | 808 | 1.13 | 0.79 |
| 64 | 1 | 0.41 | 0.28 | 0.54 | 0.52 | 794.34 | 0.71 | 209 | 946 | 667 | 806 | 1.17 | 0.78 |
| 65 | 0 | 0.72 | 0.99 | 0.45 | 0.46 | 779.18 | 0.92 | 106 | 812 | 747 | 780 | 0.32 | 0.75 |
| 66 | 0 | 0.72 | 0.95 | 0.48 | 0.48 | 785.53 | 0.89 | 92 | 835 | 739 | 787 | 0.46 | 0.76 |
| 67 | 0 | 0.69 | 0.91 | 0.46 | 0.48 | 782.16 | 0.87 | 174 | 838 | 730 | 784 | 0.51 | 0.76 |
| 68 | 0 | 0.69 | 0.94 | 0.42 | 0.45 | 771.91 | 0.88 | 80 | 822 | 725 | 773 | 0.47 | 0.74 |
| 69 | 0 | 0.68 | 0.98 | 0.37 | 0.40 | 761.58 | 0.91 | 69 | 800 | 725 | 763 | 0.37 | 0.72 |
| 70 | 0 | 0.68 | 0.92 | 0.43 | 0.45 | 773.23 | 0.88 | 57 | 826 | 724 | 775 | 0.50 | 0.74 |
| 71 | 0 | 0.68 | 0.97 | 0.39 | 0.40 | 763.67 | 0.89 | 50 | 809 | 721 | 765 | 0.43 | 0.72 |
| 72 | 0 | 0.65 | 0.83 | 0.47 | 0.48 | 783.67 | 0.85 | 83 | 853 | 720 | 787 | 0.62 | 0.76 |
| 73 | 0 | 0.64 | 0.86 | 0.41 | 0.42 | 766.47 | 0.86 | 130 | 829 | 709 | 769 | 0.58 | 0.73 |
| 74 | 0 | 0.64 | 0.87 | 0.40 | 0.40 | 764.57 | 0.86 | 114 | 826 | 708 | 767 | 0.57 | 0.72 |
| 75 | 0 | 0.62 | 0.77 | 0.48 | 0.48 | 783.52 | 0.82 | 61 | 863 | 711 | 787 | 0.70 | 0.76 |
| 76 | 0 | 0.62 | 0.93 | 0.31 | 0.32 | 744.08 | 0.88 | 152 | 795 | 697 | 746 | 0.49 | 0.68 |
| 77 | 0 | 0.61 | 0.97 | 0.24 | 0.24 | 726.96 | 0.90 | 45 | 766 | 690 | 728 | 0.39 | 0.65 |
| 78 | 0 | 0.60 | 0.82 | 0.37 | 0.37 | 759.69 | 0.84 | 54 | 828 | 697 | 763 | 0.63 | 0.71 |
| 79 | 0 | 0.59 | 0.82 | 0.36 | 0.36 | 754.42 | 0.84 | 65 | 822 | 693 | 757 | 0.63 | 0.70 |
| 80 | 0 | 0.59 | 0.92 | 0.25 | 0.26 | 729.88 | 0.87 | 55 | 781 | 682 | 732 | 0.50 | 0.66 |
| 81 | 0 | 0.59 | 0.86 | 0.32 | 0.32 | 743.41 | 0.85 | 187 | 805 | 687 | 746 | 0.59 | 0.68 |
| 82 | 0 | 0.58 | 0.74 | 0.43 | 0.42 | 770.81 | 0.82 | 212 | 854 | 696 | 775 | 0.74 | 0.73 |
| 83 | 0 | 0.58 | 1.00 | 0.15 | 0.15 | 691.76 | 0.93 | 44 | 718 | 666 | 692 | 0.29 | 0.59 |
| 84 | 0 | 0.55 | 0.79 | 0.30 | 0.32 | 741.07 | 0.83 | 64 | 814 | 674 | 744 | 0.69 | 0.68 |
| 85 | 0 | 0.55 | 0.75 | 0.32 | 0.35 | 744.54 | 0.82 | 184 | 822 | 675 | 748 | 0.71 | 0.69 |
| 86 | 0 | 0.54 | 0.94 | 0.14 | 0.15 | 689.15 | 0.88 | 22 | 734 | 647 | 691 | 0.48 | 0.59 |
| 87 | 0 | 0.54 | 0.82 | 0.26 | 0.26 | 730.40 | 0.84 | 248 | 796 | 670 | 733 | 0.63 | 0.66 |
| 88 | 0 | 0.53 | 0.77 | 0.28 | 0.30 | 736.16 | 0.83 | 241 | 810 | 669 | 740 | 0.70 | 0.67 |
| 89 | 0 | 0.53 | 0.16 | 0.90 | 0.88 | 865.92 | 0.66 | 170 | 1069 | 701 | 885 | 1.37 | 0.93 |
| 90 | 0 | 0.52 | 0.12 | 0.93 | 0.92 | 882.27 | 0.64 | 121 | 1102 | 707 | 904 | 1.43 | 0.96 |
| 91 | 0 | 0.52 | 0.88 | 0.16 | 0.17 | 695.47 | 0.86 | 283 | 750 | 645 | 697 | 0.56 | 0.60 |
| 92 | 0 | 0.52 | 0.10 | 0.94 | 0.94 | 894.36 | 0.63 | 172 | 1125 | 711 | 918 | 1.47 | 0.99 |
| 93 | 0 | 0.51 | 0.89 | 0.14 | 0.14 | 687.26 | 0.87 | 20 | 739 | 639 | 689 | 0.54 | 0.58 |
| 94 | 0 | 0.50 | 0.90 | 0.10 | 0.11 | 667.15 | 0.87 | 40 | 715 | 623 | 669 | 0.52 | 0.55 |
| 95 | 0 | 0.50 | 0.17 | 0.83 | 0.80 | 844.70 | 0.66 | 271 | 1041 | 686 | 863 | 1.36 | 0.88 |
| 96 | 0 | 0.49 | 0.18 | 0.81 | 0.77 | 836.60 | 0.67 | 205 | 1022 | 685 | 853 | 1.31 | 0.87 |
| 97 | 0 | 0.48 | 0.68 | 0.28 | 0.30 | 736.33 | 0.80 | 79 | 823 | 659 | 741 | 0.80 | 0.67 |
| 98 | 0 | 0.48 | 0.10 | 0.89 | 0.85 | 857.36 | 0.63 | 3 | 1079 | 681 | 880 | 1.47 | 0.91 |
| 99 | 0 | 0.48 | 0.95 | 0.01 | 0.01 | 608.79 | 0.88 | 288 | 647 | 573 | 610 | 0.46 | 0.46 |
| 100 | 0 | 0.47 | 0.72 | 0.22 | 0.23 | 710.96 | 0.81 | 76 | 792 | 638 | 715 | 0.77 | 0.62 |
| 101 | 0 | 0.47 | 0.03 | 0.92 | 0.88 | 868.42 | 0.60 | 263 | 1125 | 671 | 898 | 1.61 | 0.93 |
| 102 | 0 | 0.47 | 0.50 | 0.44 | 0.42 | 769.63 | 0.77 | 176 | 879 | 674 | 777 | 0.93 | 0.73 |
| 103 | 0 | 0.46 | 0.72 | 0.21 | 0.21 | 703.67 | 0.81 | 151 | 782 | 633 | 708 | 0.76 | 0.61 |
| 104 | 0 | 0.46 | 0.66 | 0.26 | 0.26 | 728.53 | 0.79 | 66 | 818 | 649 | 733 | 0.82 | 0.66 |
| 105 | 0 | 0.45 | 0.74 | 0.17 | 0.17 | 696.43 | 0.81 | 222 | 773 | 627 | 700 | 0.75 | 0.60 |
| 106 | 0 | 0.45 | 0.63 | 0.27 | 0.26 | 732.77 | 0.78 | 51 | 828 | 649 | 738 | 0.86 | 0.66 |
| 107 | 0 | 0.44 | 0.53 | 0.35 | 0.36 | 750.82 | 0.77 | 247 | 854 | 660 | 757 | 0.91 | 0.70 |
| 108 | 0 | 0.43 | 0.75 | 0.12 | 0.12 | 672.31 | 0.82 | 214 | 743 | 609 | 676 | 0.72 | 0.56 |
| 109 | 0 | 0.43 | 0.15 | 0.74 | 0.67 | 826.75 | 0.65 | 104 | 1022 | 669 | 845 | 1.38 | 0.84 |
| 110 | 0 | 0.41 | 0.72 | 0.11 | 0.12 | 670.64 | 0.81 | 84 | 747 | 602 | 675 | 0.77 | 0.56 |
| 111 | 0 | 0.41 | 0.07 | 0.80 | 0.71 | 829.18 | 0.62 | 8 | 1050 | 655 | 852 | 1.50 | 0.85 |
| 112 | 0 | 0.41 | 0.64 | 0.19 | 0.17 | 698.16 | 0.79 | 41 | 787 | 620 | 703 | 0.85 | 0.60 |
| 113 | 0 | 0.41 | 0.47 | 0.35 | 0.35 | 745.31 | 0.76 | 127 | 856 | 649 | 753 | 0.97 | 0.69 |
| 114 | 0 | 0.41 | 0.05 | 0.82 | 0.71 | 831.10 | 0.61 | 124 | 1069 | 646 | 858 | 1.58 | 0.85 |
| 115 | 0 | 0.40 | 0.56 | 0.25 | 0.24 | 723.47 | 0.78 | 100 | 822 | 637 | 729 | 0.90 | 0.65 |
| 116 | 0 | 0.40 | 0.40 | 0.41 | 0.37 | 760.05 | 0.74 | 11 | 882 | 655 | 769 | 1.03 | 0.71 |
| 117 | 0 | 0.39 | 0.66 | 0.12 | 0.12 | 673.83 | 0.79 | 203 | 756 | 601 | 678 | 0.82 | 0.56 |
| 118 | 0 | 0.39 | 0.15 | 0.65 | 0.59 | 809.28 | 0.65 | 194 | 1001 | 654 | 828 | 1.38 | 0.81 |
| 119 | 0 | 0.38 | 0.59 | 0.17 | 0.17 | 694.24 | 0.78 | 98 | 787 | 613 | 700 | 0.88 | 0.60 |
| 120 | 0 | 0.38 | 0.14 | 0.66 | 0.59 | 811.11 | 0.64 | 266 | 1011 | 651 | 831 | 1.42 | 0.81 |
| 121 | 0 | 0.38 | 0.28 | 0.49 | 0.46 | 777.15 | 0.71 | 12 | 924 | 654 | 789 | 1.17 | 0.75 |
| 122 | 0 | 0.37 | 0.30 | 0.45 | 0.42 | 769.34 | 0.71 | 101 | 913 | 648 | 781 | 1.16 | 0.73 |
| 123 | 0 | 0.37 | 0.35 | 0.39 | 0.37 | 755.94 | 0.72 | 25 | 892 | 641 | 766 | 1.12 | 0.71 |
| 124 | 0 | 0.37 | 0.41 | 0.34 | 0.32 | 742.72 | 0.74 | 285 | 861 | 641 | 751 | 1.02 | 0.68 |
| 125 | 0 | 0.37 | 0.56 | 0.18 | 0.17 | 694.81 | 0.77 | 88 | 790 | 611 | 701 | 0.90 | 0.60 |
| 126 | 0 | 0.36 | 0.49 | 0.23 | 0.23 | 709.30 | 0.76 | 287 | 813 | 619 | 716 | 0.95 | 0.62 |
| 127 | 0 | 0.35 | 0.10 | 0.63 | 0.57 | 804.51 | 0.64 | 182 | 1006 | 643 | 825 | 1.44 | 0.80 |
| 128 | 0 | 0.34 | 0.63 | 0.05 | 0.07 | 639.75 | 0.79 | 289 | 722 | 567 | 644 | 0.86 | 0.51 |
| 129 | 0 | 0.32 | 0.07 | 0.62 | 0.54 | 800.53 | 0.62 | 226 | 1014 | 632 | 823 | 1.50 | 0.79 |
| 130 | 0 | 0.32 | 0.32 | 0.33 | 0.32 | 739.28 | 0.71 | 249 | 876 | 624 | 750 | 1.14 | 0.68 |
| 131 | 0 | 0.32 | 0.56 | 0.08 | 0.08 | 654.02 | 0.77 | 225 | 743 | 576 | 659 | 0.90 | 0.53 |
| 132 | 0 | 0.30 | 0.14 | 0.50 | 0.45 | 773.22 | 0.64 | 279 | 964 | 620 | 792 | 1.42 | 0.74 |
| 133 | 0 | 0.30 | 0.39 | 0.21 | 0.21 | 699.69 | 0.73 | 24 | 817 | 599 | 708 | 1.06 | 0.61 |
| 134 | 0 | 0.28 | 0.43 | 0.13 | 0.14 | 680.12 | 0.75 | 291 | 787 | 588 | 687 | 1.01 | 0.57 |
| 135 | 0 | 0.28 | 0.47 | 0.09 | 0.10 | 660.41 | 0.76 | 53 | 759 | 575 | 667 | 0.97 | 0.54 |
| 136 | 0 | 0.27 | 0.26 | 0.29 | 0.26 | 729.65 | 0.70 | 21 | 872 | 611 | 741 | 1.20 | 0.66 |
| 137 | 0 | 0.25 | 0.46 | 0.04 | 0.06 | 635.96 | 0.75 | 276 | 733 | 552 | 642 | 0.99 | 0.50 |
| 138 | 0 | 0.25 | 0.08 | 0.46 | 0.37 | 760.09 | 0.63 | 265 | 960 | 602 | 781 | 1.49 | 0.71 |
| 139 | 0 | 0.23 | 0.14 | 0.34 | 0.30 | 734.75 | 0.65 | 72 | 910 | 593 | 752 | 1.39 | 0.67 |
| 140 | 0 | 0.22 | 0.39 | 0.05 | 0.06 | 638.42 | 0.74 | 116 | 743 | 549 | 646 | 1.04 | 0.50 |
| 141 | 0 | 0.22 | 0.26 | 0.19 | 0.15 | 690.95 | 0.70 | 32 | 823 | 580 | 702 | 1.18 | 0.59 |
| 142 | 0 | 0.21 | 0.34 | 0.08 | 0.08 | 651.42 | 0.72 | 33 | 769 | 552 | 661 | 1.13 | 0.52 |
| 143 | 0 | 0.20 | 0.17 | 0.23 | 0.21 | 705.05 | 0.66 | 103 | 869 | 572 | 721 | 1.36 | 0.61 |
| 144 | 0 | 0.20 | 0.12 | 0.30 | 0.24 | 725.47 | 0.64 | 301 | 906 | 581 | 744 | 1.43 | 0.65 |
| 145 | 0 | 0.19 | 0.35 | 0.03 | 0.04 | 624.23 | 0.72 | 19 | 736 | 529 | 633 | 1.12 | 0.48 |
| 146 | 0 | 0.16 | 0.02 | 0.37 | 0.24 | 722.84 | 0.52 | 195 | 1000 | 523 | 761 | 1.91 | 0.65 |
| 147 | 0 | 0.15 | 0.26 | 0.03 | 0.05 | 629.49 | 0.70 | 34 | 753 | 526 | 640 | 1.20 | 0.49 |
| 148 | 0 | 0.10 | 0.04 | 0.20 | 0.14 | 683.70 | 0.60 | 179 | 883 | 529 | 706 | 1.60 | 0.58 |
| 149 | 0 | 0.10 | 0.10 | 0.10 | 0.08 | 653.33 | 0.63 | 36 | 822 | 520 | 671 | 1.47 | 0.53 |
| 150 | 0 | 0.07 | 0.02 | 0.15 | 0.10 | 660.46 | 0.52 | 85 | 914 | 477 | 696 | 1.91 | 0.54 |
| 151 | 0 | 0.07 | 0.12 | 0.01 | 0.01 | 597.89 | 0.64 | 295 | 746 | 479 | 613 | 1.43 | 0.44 |
| 152 | 0 | 0.06 | 0.06 | 0.06 | 0.05 | 630.69 | 0.61 | 292 | 804 | 495 | 649 | 1.54 | 0.49 |
| 153 | 0 | 0.03 | 0.05 | 0.00 | 0.00 | 536.97 | 0.60 | 299 | 691 | 418 | 554 | 1.58 | 0.36 |
| 154 | 0 | 0.03 | 0.00 | 0.07 | 0.03 | 616.36 | 0.51 | 300 | 860 | 442 | 651 | 1.94 | 0.47 |
| 155 | 0 | 0.02 | 0.01 | 0.06 | 0.01 | 613.35 | 0.52 | 296 | 855 | 440 | 647 | 1.93 | 0.46 |
| 156 | 0 | 0.02 | 0.03 | 0.02 | 0.01 | 607.80 | 0.57 | 17 | 804 | 460 | 632 | 1.71 | 0.46 |

Columns include: Rank (final position), Eligible (1 = genotype meeting HSI ≤ 1.3 and median thresholds for MP and STI; 0 = otherwise), HT_score (composite score), d_HSI, d_MP, d_STI (percentile-based desirability values for each index), GMP (geometric mean productivity), YSI (yield stability index), Gen (genotype identifier), YP (grain yield under normal conditions), YS (grain yield under stress), MP (mean productivity), HSI (heat susceptibility index), and STI (stress tolerance index).

**References**

Khodaee, Sayyed Mohammad Mehdi, Maryam Hashemi, Aghafakhr Mirlohi, Mohammad Mahdi Majidi, Sivakumar Sukumaran, Mohsen Esmaelzaeh Moghaddam, and Mohammad Abdollahi. 2021. “Root Characteristics of an Elite Spring Wheat Panel under Contrasting Water Treatments and Their Genome-Wide Association Study.” *Rhizosphere* 19(July):100413. doi: 10.1016/j.rhisph.2021.100413.

Kumar, Pradeep, Vikas Gupta, Gyanendra Singh, Charan Singh, Bhudeva S. Tyagi, and Gyanendra Pratap Singh. 2021. “Assessment of Terminal Heat Tolerance Based on Agro-Morphological and Stress Selection Indices in Wheat.” *Cereal Research Communications* 49(2):217–26. doi: 10.1007/s42976-020-00112-2.

Lopes, M. S., S. Dreisigacker, R. J. Peña, S. Sukumaran, and M. P. Reynolds. 2015. “Genetic Characterization of the Wheat Association Mapping Initiative (WAMI) Panel for Dissection of Complex Traits in Spring Wheat.” *Theoretical and Applied Genetics* 128(3):453–64. doi: 10.1007/s00122-014-2444-2.

Sharma, Surakshya, Eishaina Chaudhary, Pratik Gautam, Rashmi Poudel, Sushma Sapkota, Sweksha Ghimire, Bibisha Timalsina, Puja Roka, Kriti Bhattarai, Manoj Pariyar, Kapil Neupane, Anil Aryal, Ganesh G.C, Mukti Ram Poudel, and Radhakrishna Bhandari. 2023. “Identification of Heat Stress Tolerant Wheat Genotype Using Stress Tolerance Indices.” *Journal of Soil, Plant and Environment* 2(2):16–27. doi: 10.56946/jspae.v2i2.185.

Sukumaran, Sivakumar, Susanne Dreisigacker, Marta Lopes, Perla Chavez, and Matthew P. Reynolds. 2015. “Genome-Wide Association Study for Grain Yield and Related Traits in an Elite Spring Wheat Population Grown in Temperate Irrigated Environments.” *TAG. Theoretical and Applied Genetics. Theoretische Und Angewandte Genetik* 128(2):353–63. doi: 10.1007/S00122-014-2435-3.
